# The alfalfa U-box E3 ligase MsPUB210 interacts with MsICE1 and positively regulates cold tolerance in transgenic Arabidopsis

**DOI:** 10.3389/fpls.2026.1772992

**Published:** 2026-03-13

**Authors:** Meng Wang, Shuaixian Li, Xiaoyue Zhu, Meiyan Guo, Changhong Guo, Yongjun Shu

**Affiliations:** Key Laboratory of Molecular Cytogenetics and Genetic Breeding of Heilongjiang Province, College of Life Science and Technology, Harbin Normal University, Harbin, China

**Keywords:** afalfa, cold tolerance, GRN, AlphaFold 2, ubiquitination, U-box E3 ubiquitin ligases

## Abstract

**Introduction:**

Cold stress severely restricts the productivity of Alfalfa (*Medicago sativa L.*), prompting the evolution of complex resistance mechanisms involving post-translational modifications. In this study, we characterized *MsPUB210*, a gene encoding a U-box E3 ubiquitin ligase, to elucidate its role in cold adaptation.

**Methods:**

Phylogenetic analysis confirmed that MsPUB210 is homologous to *Arabidopsis* U-box proteins, and gene regulatory network (GRN)-guided protein-protein interaction (PPI) network analysis, together with co-expression investigation, first predicted a potential association between *MsPUB210* and the transcription factor MsICE1. This prediction was further corroborated by AlphaFold2 (AF2)-predicted 3D structures of their heteromeric complexes, supporting the biological plausibility of this interaction. We demonstrated via yeast two-hybrid (Y2H) assays that MsPUB210 physically interacts with the transcription factor MsICE1. Physiological and biochemical profiling was quantified by measuring chlorophyll (CHL) content, malondialdehyde (MDA) accumulation, proline (Pro) levels, and the activities of catalase (CAT), peroxidase (POD), and superoxide dismutase (SOD).

**Results:**

Heterologous overexpression of *MsPUB210* in *Arabidopsis thaliana* significantly enhanced cold tolerance, characterized by reduced reactive oxygen species (ROS) accumulation and mitigated lipid peroxidation compared to wild-type plants.

**Discussion:**

Furthermore, *MsPUB210* was found to upregulate downstream cold-responsive genes, suggesting it functions as a positive regulator within the ICE-CBF-COR signaling cascade. Collectively, these findings highlight the pivotal role of *MsPUB210* in ubiquitin-mediated cold signaling and identify it as a promising genetic target for breeding cold-resilient forage crops.

## 1 Introduction

Plants frequently encounter environmental stress, among which low temperature is one of the most common damaging factors. Low temperatures disrupt membrane stability and impair enzyme activities. Cold reduces photosynthetic efficiency, leading to metabolic imbalance and cellular damage. Constant exposure to various environmental conditions and temperature stress presents one of the most significant challenges to a plant’s growth, development, and overall productivity (Shu and Yang, 2017). Low temperatures can cause cellular dysfunction and even irreversible tissue damage by damaging membrane integrity, inhibiting enzyme activity, and reducing photosynthetic efficiency. In-depth elucidation of plant cold tolerance mechanisms is crucial for improving crop yield and quality and enhancing the stress resilience of perennial forage crops. Alfalfa (*Medicago sativa L.*) is a top-tier legume in agricultural systems that combines high yield potential with marked sensitivity to chilling injury in temperate regions (Zhou et al., 2024).

Due to their sessility, plants have developed multiple regulatory mechanisms to mitigate cold stress (Wang et al., 2023a), including transcriptional, translational, and post-translational mechanisms—enabling them to mitigate cold stress by perceiving and transmitting internal or external signals (Shu and Yang, 2017). Plants can quickly and reversibly alter protein stability and activity in response to environmental stimuli thanks to post-translational modification (PTM), which enables modifications during various plant-environment interactions (Wilson et al., 2016), serving as a pivotal regulatory mechanism. Of the various PTMs, phosphorylation, SUMOylation, acetylation and ubiquitination (Ub)—often occurring after phosphorylation—emerge as some of the most versatile and tightly regulated mechanisms (Li et al., 2023b). From protein degradation to activation of signaling pathways, Ub controls a wide range of processes (Vierstra, 2009; Al-Saharin et al., 2022; Karthik et al., 2024). How plants sense and adapt to cold stress is crucial for improving the resilience of perennial forage crops such as alfalfa, a high-valued legume in agronomy ines high yield potential with marked sensitivity to chilling injury in temperate regions (Zhou et al., 2024).

Ubiquitination regulates the stability, activity, and functions of proteins by conjugating a small conserved regulatory protein consisting of 76 amino acids to lysine or N-terminal on substrate proteins through an isopeptide bond (Suryadinata et al., 2014; Zhang et al., 2024). The sequential enzymatic cascade powering the ubiquitin-proteasome system (UPS) includes the ubiquitin-activating enzyme (E1), the ubiquitin-conjugating enzyme (E2), and the ubiquitin ligase (E3) (Hershko and Ciechanover, 1998; Zhang et al., 2024). After being combined with an E2 conjugating enzyme, the activated ubiquitin cooperates with an E3 ubiquitin ligase to mediate the final covalent attachment between the substrate protein and ubiquitin to complete the ubiquitination process (Mevissen and Komander, 2017; Yang et al., 2021). According to Komander et al., this attachment may cause degradation, encourage protein-protein interactions, affect subcellular localization, or control signaling pathways (Komander and Rape, 2012; Ej et al., 2020). E3 ligases play an indispensable role in the ubiquitination network, converting upstream signals into precise regulatory outcomes, especially during stress adaptation. In a key-lock manner, they physically recognize lysine, serine, threonine, or cysteine residues as unique targets (Zheng and Shabek, 2017). The functional diversity of E3 ligases thus provides a molecular basis for the selectivity and specificity of ubiquitin signaling (Ardley and Robinson, 2005; Vierstra, 2009; Kelley and Estelle, 2012).

Plant genomes encode thousands of E3 ligases, reflecting the remarkable complexity of plant signaling and environmental adaptation. This vast diversity of E3 ligases makes them the most expanded component of the ubiquitin-proteasome system (Hwang et al., 2016; Liu et al., 2021). Based on their structural and mechanistic features, E3 ligases can be classified into four major families: RING, HECT, U-box, RING-IBR-RING (RBR) (Buetow and Huang, 2016; Yang et al., 2021). While HECT-type ligases create a covalent intermediate complex with ubiquitin prior to transferring the ubiquitin to target protein, RING-type ligases transfer ubiquitin directly from E2 to the substrate. Structurally similar to RING ligases, U-box ligases stabilize their catalytic conformation via hydrogen-bond networks rather than zinc ions, enabling them to function well in stressful environments that perturb redox homeostasis (Lyzenga and Stone, 2012; Zhang et al., 2024). The recently discovered RING-IBR-RING (RBR) E3 ligases use linear ubiquitin chains to ubiquitinate substrates, with an assembly complex consisting of HOIP (Yang et al., 2021).

Based on previous studies, the E3 ligase families—RING, HECT and U-box—all contribute to temperature adaptation by modulating the abundance and activity of critical transcription factors and stress-related proteins (Miura et al., 2007; Stone, 2014; Ryu et al., 2019; Meng et al., 2023). In wheat, *TaSDIR1-4A* enhances drought resistance through a distinct mechanism. It mediates the polyubiquitination and subsequent proteolysis of specific C-terminal residues, which in turn activates TaWRKY29. Activated TaWRKY29 binds directly to *TaABI5*’s promoter region, promoting transcription and strengthening stress signaling pathways dependent on abscisic acid (ABA) (Walczakl et al., 2012; Zhang et al., 2017; Pao et al., 2018).

U-box type E3 ubiquitin ligases (PUBs) are widely conserved across plant species, with a motif of approximately 70 amino acids. Plant U-box domains contain tetratricopeptide repeats (TPR) and armadillo repeats (ARM), which have an impact on protein-protein interactions. They transfer ubiquitin to important regulatory proteins that control stress-responsive signaling pathways (Trujillo, 2018) and are involved in signal transduction (Liu et al., 2024). Early genome-wide analysis revealed that GhPUB85A and GhPUB45D in cotton mediate defense responses through their tetratricopeptide repeat (TPR) domains, which are structurally similar to those of AtCHIPs, suggesting a conserved mechanism of substrate recognition under stress (Yan et al., 2003; Lu et al., 2020). Functional analyses shed light on the mechanistic roles of U-box E3 ligases in regulating abiotic stress tolerance (Sherpa et al., 2022). In rice (*Oryza sativa*), salt and cold stress induce transcription of members of the OsPUBs. Liu et al. discovered that AtPUB19 is a negative regulator in ABA-mediated stress signaling in *Arabidopsis thaliana*, connecting PUB ligase activity to stomatal regulation pathways (Liu et al., 2011), implying that these genes may serve a variety of functions in response to abiotic and biotic challenges, such as enhancing cell membrane stability and survival. Similarly, the apple homolog *MdPUB23* reduces cold tolerance by targeting *MdICE1* (Liu et al., 2011), which indicates that these genes may serve a wide range of functions in coping with abiotic and biotic stresses, for instance, by enhancing cell membrane stability and survival. In contrast, *TaPUB1* has been characterized as a positive regulator governing drought tolerance in wheat, primarily by lowering reactive oxygen species (ROS) accumulation, maintaining redox homeostasis, and mitigating the oxidative damage in response to drought stress (Zhang et al., 2017). The evolutionary divergence of PUBs has been demonstrated by the temporally distinct ubiquitination of ICE1 by AtPUB25 and AtPUB26 proteins in *Arabidopsis thaliana*, which is an example of dynamic modulation of cold-response transcription through E3 chain topology switching (Wang et al., 2022). Through the activation and regulation of CBF-dependent transcriptional programs, this dual-phase ubiquitination process aids in adjusting the intensity and duration of the cold signaling response, further highlighting how PUBs can function differently across species (Wang et al., 2019, 2023b).

Nonetheless, in *Medicago sativa*, the genetic elements that regulate the spatiotemporal expression of cold-related factors, as well as their activation and the molecular mechanism, remain largely unknown. Our genome-wide analyses have identified 210 PUB genes in alfalfa, indicating a high level of diversification that may be connected to environmental adaptability (Li et al., 2024). This investigation suggests that the cold-induced MsPUB210 protein may stabilize ICE1-like factors to modulate CBF-related pathways, thereby acting as a positive regulator in the ubiquitin-mediated cold signaling cascade.

We investigated whether cold-induced upregulation of unique alfalfa U-box E3 ligases contributes to chilling tolerance in *Medicago sativa* and how they interact with cold-related factors. We first confirmed its function as a cold-tolerance candidate gene via gene family analysis; then validate the feasibility of its heterologous expression in *Arabidopsis thaliana* and predicted their functional roles in cold stress response pathway of *Medicago sativa* through GRN analysis and with AlphaFold2 (AF2) structural prediction. Coding sequences from *Medicago sativa* were cloned and heterologously expressed in *Arabidopsis thaliana* to interrogate their in-plant activities. Transgenic lines were assessed using complementary physiological and molecular readouts. Quantitative expression analysis of canonical cold markers (e.g., *CBF3*, *COR47*, *RAB18*) in Medicago sativa was performed to assess downstream signaling responses (Li et al., 2024). By integrating phenotypic, biochemical, and transcriptional data, this study attempted to ascertain whether the upregulation of MsPUB genes improves chilling tolerance through mechanisms similar to those reported in model species by combining phenotypic, biochemical, and transcriptional data. The results provide mechanistic insights into ubiquitin-mediated cold adaptation and offer potential genetic targets for the breeding of cold-resilient forage cultivars.

## 2 Materials and methods

### 2.1 Bioanalysis and protein-protein interaction validation

#### 2.1.1 Construction of PPI networks for cold-responsive GRN analysis

To further explore the functional association of *MsPUB210* and *MsICE1* genes in the cold-responsive GRN (Gene regulatory network), a PPI network was constructed using the STRING database (https://string-db.org) with *Arabidopsis thaliana* as the background organism. The input sequences included two core proteins (MsPUB210 and MsICE1) and 25 previously validated cold stress-related protein sequences from TAIR database (https://www.arabidopsis.org/). This design integrated co-expression evidence and direct protein-binding predictions to provide a basis for analyzing core regulatory relationships in the cold-responsive GRN, as well as theoretical support for designing and interpreting subsequent transgenic and protein interaction experiments. Additionally, to verify cross-species conservation of the regulatory relationship in GRN, another PPI network focusing on AtICE1 and MsPUB210 proteins were constructed. For all network constructions, the interaction confidence score was set to low confidence (0.15) to retain more potential GRN-related clues, the full STRING network was used, and the maximum number of interactions per shell was specified as 10. All resulting PPI networks (reflecting GRN-related associations) were visualized using Cytoscape_v3.10.4. To investigate the functional association between *MsPUB210* and *MsICE1* at the transcriptional level, RNA-seq data from multiple alfalfa cultivars were analyzed using Salmon (version 0.12.0) (Patro et al., 2017) for transcript quantification. The transcripts per million (TPM) values of *MsPUB210* and *MsICE1* were calculated to normalize gene expression across samples. Pearson’s correlation coefficient (r) and corresponding statistical significance (p-value) then were displayed by R software (version 4.5.2), to quantify the strength and significance of the co-expression relationship between the two genes. Multiple sequence alignment was performed using the ClustalW algorithm. A neighbor-joining (NJ) tree was subsequently built in MEGA11 software, with 1000 bootstrap replicates to validate the reliability of phylogenetic clustering. To further validate the cross-species conservation of PUB*-*ICE1 interaction, PUB and ICE1 homologous protein sequences from seven additional plant species were collected, including *Arabidopsis thaliana*, *Oryza sativa subsp. japonica*, *Capsicum annuum*, *Trifolium pratense*, *Nicotiana attenuata*, *Glycine max*, and *Setaria italica*. These sequences were input into STRING Tools (for multiple protein sequences) with species-specific background databases.

#### 2.1.2 AlphaFold2 analysis for protein interaction prediction and *MsPUB210* transformation control

To validate the predicted interactions between MsPUB210 and both MsICE1/AtICE1, thereby supporting subsequent yeast two-hybrid (Y2H) assays, we performed in silico validation using the protein_base_default model in AlphaFold2 (AF2)——a general-purpose core model optimized based on the Evoformer architecture for complex prediction. To assess cross-species conservation, we picked eight species (*A. thaliana*, *O. sativa subsp. japonica*, *C. annuum*, *T. pratense*, *N. attenuata*, *G. max*, *S. italica*, and *M. domestica*) for cross-species conservation analysis. Subsequently, potential ubiquitination sites (lysine residues, K) located at PUB-ICE1 interaction interfaces were identified using PLIP (Schake et al., 2025) and LigPlus (version 2.3.1) (Laskowski and Swindells, 2011) tools. The key computational parameters were differentiated: For core interaction pairs (MsPUB210-MsICE1, MsPUB210-AtICE1): 5 random seeds and 5 iterative refinement cycles for initial model generation, 200 maximum iterations for structural relaxation, and a greedy pairing strategy. For cross-species PUB-ICE1 pairs: 1 random seed (with all other parameters identical to the core pairs) to efficiently assess the structural conservation of interspecies interactions. The complex protein structures were visualized, and interaction interfaces were analyzed using UCSF ChimeraX (version 1.11) and PyMOL (version 2.0.4). Prior to the Y2H assay, a transformation control assay was conducted to ensure the reliability of experimental conditions. For Y2H construct preparation, the 783-bp full-length coding sequence of *MsPUB210* was amplified by PCR and subcloned into the pGBKT7 vector via homologous recombination, generating a fusion construct in which *MsPUB210* was linked to the GAL4 DNA-binding domain. Primers of AD/BD vectors were listed in **Supplementary Table 1**. Separately, three groups—the positive control, empty AD/empty BD vector combination, and empty AD*/MsPUB210*-BD vector combination—were transformed into *Saccharomyces cerevisiae* AH109, in accordance with the operation manual of Coolaber AH109 Yeast Competent Cells. Transformed yeast cells were first spotted on SD/-Leu/-Trp medium for the primary screening. Single colonies from the primary screening were further streaked onto SD/-Leu/-Trp/-His triple-dropout plates containing 5 mM 3-amino-1,2,4-triazole (3-AT) as a background restriction to assess autonomous transcriptional activity. Yeast cell suspensions were spotted on the selection media and cultured at 30 °C for 3 to 5 days. Yeast growth on SD/-Leu/Trp/-His with 3-AT (5 mM) was interpreted as evidence of auto-activation. Each experiment was conducted with at least three biological replicates, with three technical replicates per biological replicate.

#### 2.1.3 Y2H assay

To verify the physical interaction between MsPUB210 and MsICE1, a yeast two-hybrid (Y2H) assay was performed. Complete recombination of *MsPUB210* and *MsICE1* was subcloned into the pGKBT7 or pGADT7 vector in fusion with BD and AD respectively, to create the bait and prey constructs. The target constructions were co-transformed into the yeast strains AH109 following the manufacture’s protocol. The transformed yeast cells were plated onto selective medium (SD/-Leu/-Trp) for initial selection to confirm the successful transformation. Single colonies were then transferred onto high-stringency selective medium SD/-Ade/-Trp/-Leu/-His medium containing 5 mM 3-AT to assess protein-protein interactions. Yeast cells were incubated at 30°C for 5 days, and the assay was performed with three biological replicates and each replicate with three technical replicates.

### 2.2 Genes cloning and combinations PCR-validation

Total RNA of cold-treated *Medicago sativa* leaves was isolated by RNAprep Pure Plant kit (Tiangen Biotech, Beijing, China). Using NanoDrop 2000 spectrophotometer (Thermo Fisher Scientific, Waltham, MA, USA) to concentrate and purify the RNA. To assess the integrity and quality of RNA, the Agilent 2100 Bioanalyzer (Agilent Technologies, Santa Clara, CA, USA) combined with agarose gel electrophoresis was used. First-strand cDNA was synthesized with the PrimeScript RT Kit (Toyobo, Shanghai, China). Full-length sequences of *MsPUB210* and *MsICE1* were amplified by PCR with the primer pairs listed in **Supplementary Table 2**. After PCR product purification, the amplicons were ligated into the pMD18-T vectors (TaKaRA), and Sanger sequencing was used to verify the target sequences’ accuracy.

### 2.3 Plant materials and growth conditions

#### 2.3.1 Preparation for seeds

*Medicago sativa* L. cv. Zhaodong was the experimental material in this study. The seeds were obtained from Barenbrug China Ltd. Com. (Beijing, China). After being surface sterilized with previous methods (Liu et al., 2016), seeds were sown in a substrate mixture of soil and vermiculite in a specific volumetric ratio (Li et al., 2023a).

#### 2.3.2 Germination and seedlings cultivation

During germination, each spot was covered with a transparent plastic film to maintain their stable moisture and cultivated in a controlled greenhouse under the following photoperiod and temperature conditions: 16 h of fluorescent white light (100 μE·m^−2^·s^−1^) at 24 °C, followed by 8 h of darkness at 18 °C. Throughout the seedling cultivation phase, all plants were irrigated once per week with half-strength Hoagland’s nutrient solution (Hoagland and Arnon, 1950). Wild-type *Arabidopsis thaliana* (ecotype Col-0) plants were grown under normal growth conditions as transgenic recipients until flowering.

#### 2.3.3 Sample collection and stress treatment

Seedlings were exposed to 4 °C to simulate natural cold stress conditions, and the experiment was designed with three biological replicates and three technical replicates each. Before cold treatment, triplicate control samples were quickly frozen in liquid nitrogen and stored at -80 °C for further analysis of physiological indices and gene expression levels (Li et al., 2025).

### 2.4 Agrobacterium-mediated Arabidopsis transformation and transgenic line validation

#### 2.4.1 Vector construction

The recombinant plasmids pMD18T-*MsPUB210* and pMD18T-*MsICE1*, as well as the binary vector pCAMBIA1300, were all double-digested with *BamHI* and *SalI*, and the target fragments were then subcloned into pCAMBIA1300 using T4 DNA ligase. The default CaMV 35S promoter inherent to the pCAMBIA1300 vector was directly used to drive the expression of the target genes (**Supplementary Figure 1**).

#### 2.4.2 Transformation experiment

After obtaining the recombinant overexpression constructs, transformed the recombinants into the GV3101 via the freeze-thaw transformation. Aimed at getting stable transgenic Arabidopsis, the floral dipping protocol was used. As the transformation was done, about 5–6 weeks later, the decapitation was performed to simulate the development of lateral flowers, and the immature siliques were removed to reduce contamination by WT seeds. To sterilize the surface of T0 seeds, 75% ethanol was used for 30–60 seconds, then 20% bleach (1% sodium hypochlorite) with 0.05% Tween 20 for 5 minutes. Rinse with sterile water 5–6 times to remove the bleach. All the seeds were stratified at 4°C for 2 days in darkness to break dormancy. The activated seeds were promptly placed in a growth chamber under conditions of 24°C, 60% relative humidity, and a 16 h light/8 h dark photoperiod (Clough and Bent, 1998). For transgenic seed screening, seeds were suspended in 0.1% sterile agarose and spotted on solid MS medium containing 50 mg/L kanamycin; the seedling density was approximately 3000 seeds per 150x15 mm^2^ plate.

#### 2.4.3 Identification of T3 positive lines

For further phenotypic analysis, two-week-old transformants displaying normal growth phenotypes were carefully transplanted into pots filled with a nutrient soil mixture. As T1-resistant plants grown to maturity, T2 seeds were harvested separately from each individual T1 line. T2 and T3 seeds were sterilized and planted on kanamycin-supplemented MS medium as described above. Arabidopsis DNA from infected leaves was used for PCR validation; the primer was listed in **Supplementary Table 3**, and the PCR results that matched expected sizes were sent to BGI Genomics for sequencing confirmation. Both 4-week-old wild-type (WT) and T3 transgenic plants were subjected to cold treatment in a chilling chamber, where they were incubated at 4 °C for 24 hours. All experiments were performed with three biological replicates and three technical replicates per biological replicate, and their phenotypic changes were documented systematically.

### 2.5 Quantitative expression profiling of resistance-associated genes in transgenic Arabidopsis

#### 2.5.1 *MsPUB210* RNA extraction, DNA synthesis, and qRT-PCR for cold-related genes

Set the non-stressed groups and stress-treated groups for all four-week-old seedlings of WT and transgenic *Arabidopsis thaliana*. Total RNAs were extracted and transcribed to cDNAs by the PrimeScript RT kit (Toyobo, Shanghai, China). SYBR Premix Ex Taq™ II (Toyobo, Shanghai, China) and a LightCycler 96 system were used in the quantitative real-time PCR for genes directly related to cold stress (*AtERD10*, *AtCBF3*, *AtCOR3*, *AtCOR6.6*, *AtCOR15A*, *AtCOR47*) or indirectly involved in low-temperature stress via ABA signaling (*AtRAB18*, *AtNCED3*). Glyceraldehyde-3-phosphate dehydrogenase (*GAPDH)* served as the reference gene to normalize gene expression levels. Each qPCR was performed in a 20 μL volume, consisting of 10 μL SYBR Green qPCR Master Mix, 0.8 μL primer mix, 1 μL cDNA template and 8.2 μL ddH2O). The cycling program in qPCR experiment was as follows: pre-denaturation at 95 °C for 2 min 30 s, followed by 40 cycles of 55 °C for 30 s and 72 °C for 1 min. The 2^−ΔΔCt^ algorithm was adopted to compute the relative expression levels of target genes. Each sample was assayed with three biological replicates and three technical duplicates (Liu et al., 2021). The primers of all related genes were presented in **Supplementary Table 4**.

### 2.6 Physiological and biochemical profiling of *MsPUB210*-transgenic Arabidopsis

Four to six-week-old soil-grown plants in similar stages were divided into two groups under standardized growing conditions. The control group was kept under normal conditions, while the experimental group received 4°C cold stress for 24 hours under a 16/8 light/dark cycle. For physiological and molecular characterization, mature leaves were sampled and preserved as previously described. We further measured a series of key indices: chlorophyll (CHL), proline (Pro) accumulation, malondialdehyde (MDA) levels, enzymatic activities of catalase (CAT), peroxidase (POD), and superoxide dismutase (SOD) levels. Total chlorophyll was extracted using 80%/95% ethanol or acetone and quantified spectrophotometrically. Proline content was quantitatively determined using the acid-ninhydrin colorimetric assay. MDA content was measured via the TBA assay, monitoring the decomposition rate of H_2_O_2_ to measure the CAT. POD activity was assayed using the guaiacol oxidation method, and SOD activity was evaluated via the nitroblue tetrazolium (NBT) photoreduction inhibition assay (Weydert and Cullen, 2010). All samples included three biological replicates and three technical replicates, and data are presented as means ± standard deviation (SD).

## 3 Results

### 3.1 Gene regulatory network analysis of *MsPUB210* and the AF2-predicted complex

**Figure 1:**
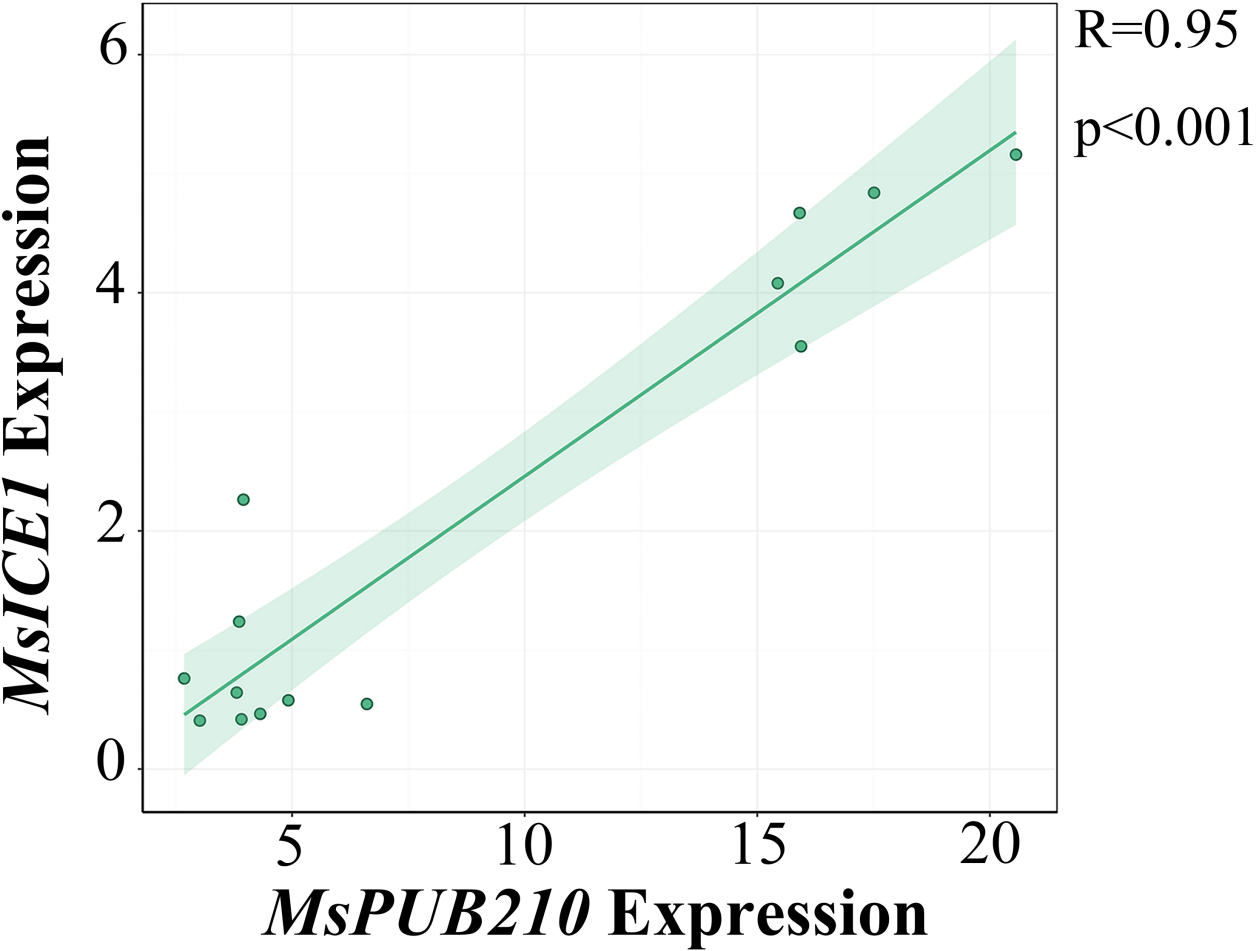
Trend line-scatter plot of co-expression between *MsICE1* and *MsPUB210*. Visualizes the transcriptional expression levels of *MsICE1* (y-axis) and *MsPUB210* (x-axis), derived from RNA-seq data of multiple alfalfa samples.

**Figure 2:**
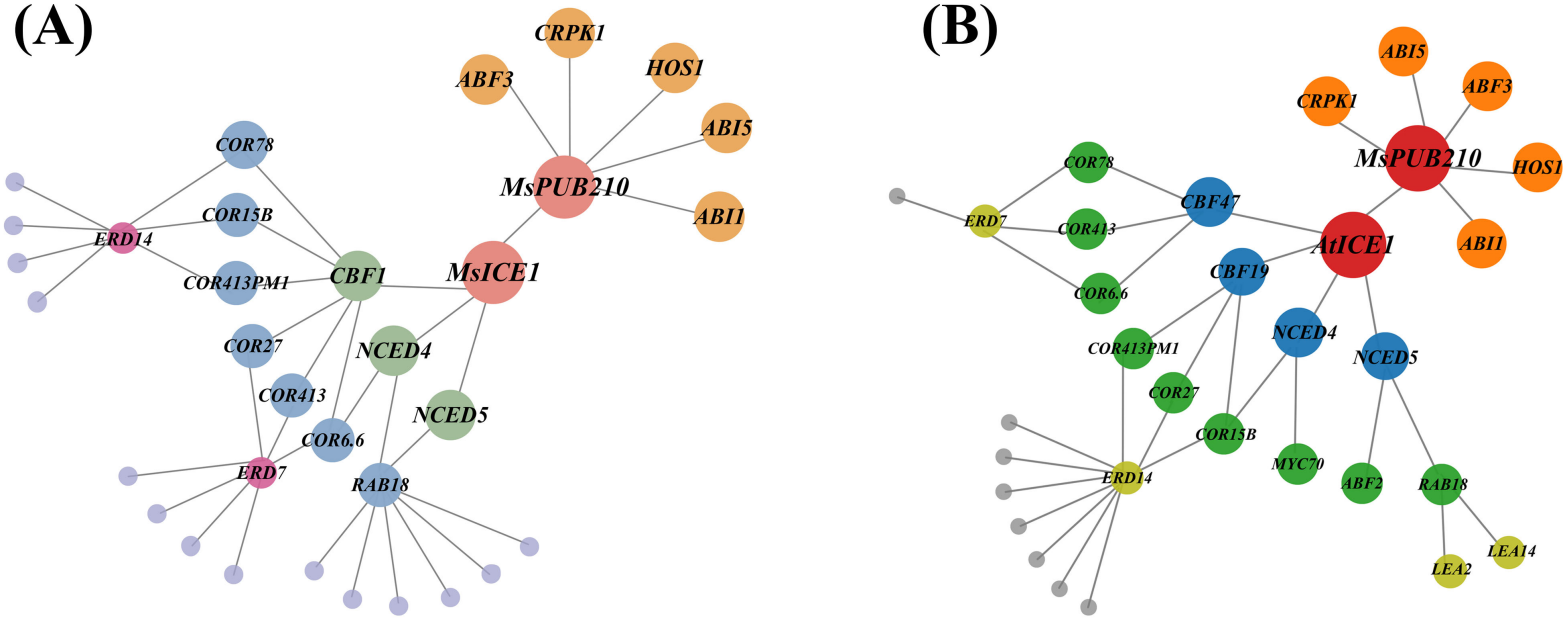
Cold-responsive gene regulatory network (GRN) centered on *MsPUB210* and *ICE1* orthologs. Light gray nodes represent downstream genes targeted by the hub. **(A)** *MsICE1*-*MsPUB210*-centered GRN: 22 nodes (proteins) and 38 edges (predicted interactions). *MsPUB210* (pink, top right) and *MsICE1* (pink) are core regulatory hubs. Light purple nodes represent downstream genes modulated by the hub. **(B)** *AtICE1*-*MsPUB210* GRN: 25 nodes and 42 edges. *MsPUB210* (red, top right) and *AtICE1* (red) are core hubs.

We evaluated the co-expression relationship between *MsPUB210* and *MsICE1* via the trend line and scatter plot (**Figure 1**). To delineate the functional role of *MsPUB210* and *MsICE1* in the cold-responsive gene regulatory network (GRN), we constructed protein-protein interaction (PPI) networks via the *Arabidopsis thaliana* STRING database, integrating these two core proteins and their co-expressed cold-responsive partners. The analysis revealed a strong positive co-expression correlation between the two genes, with R = 0.95 and a highly significant p-value (p<0.001), the raw data are presented in **Supplementary Table 5**. Consistently, in the *MsICE1*-centered network (**Figure 2A**), *MsPUB210* directly regulates *MsICE1* (homolog of *A. thaliana* At3g26744), as well as between MsPUB210 proteins and several ICE1-like proteins, reflect the potential synergistic regulatory relationship of their encoding genes in the GRN. Additionally, MsICE1 proteins are predicted to form direct connections with DREB1B (CBF1) and NCED family proteins (NCED4, NCED5)—both well-documented downstream regulators in plant cold-stress response pathways. Notably, prior studies have demonstrated that AtICE1 proteins interact with PUB proteins (Yao et al., 2017; Wang et al., 2019). We further constructed an additional GRN focusing on *MsPUB210* and *AtICE1*, which also predicted a direct interaction between these two proteins. In this network, AtICE1 is linked to multiple cold-responsive factors, including DREB family members (CBF47, CBF19) and COR proteins (COR78, COR413, COR6.6, COR27, COR15B), as shown in **Figure 2B**. All genes predicted to interact with *MsICE1* or *MsPUB210* (and their homologous counterparts) in these GRNs were selected as candidate targets for qRT-PCR analysis in transgenic plants. Specifically, we chose *AtRAB18*, *AtCOR6.6*, *AtCOR15A*, *AtCOR47*, *AtCBF3*, *AtNCED3*, and *AtERD10*—each of these genes shares homology with the cold-responsive factors identified in the aforementioned networks. Furthermore, evidence from both published studies and PPI predictions consistently supports functional interactions between PUB family proteins and ICE1 proteins across multiple species (**Supplementary Table 6**).

### 3.2 Protein interaction prediction via AlphaFold2 and validation of *MsPUB210* transformation control

**Figure 3:**
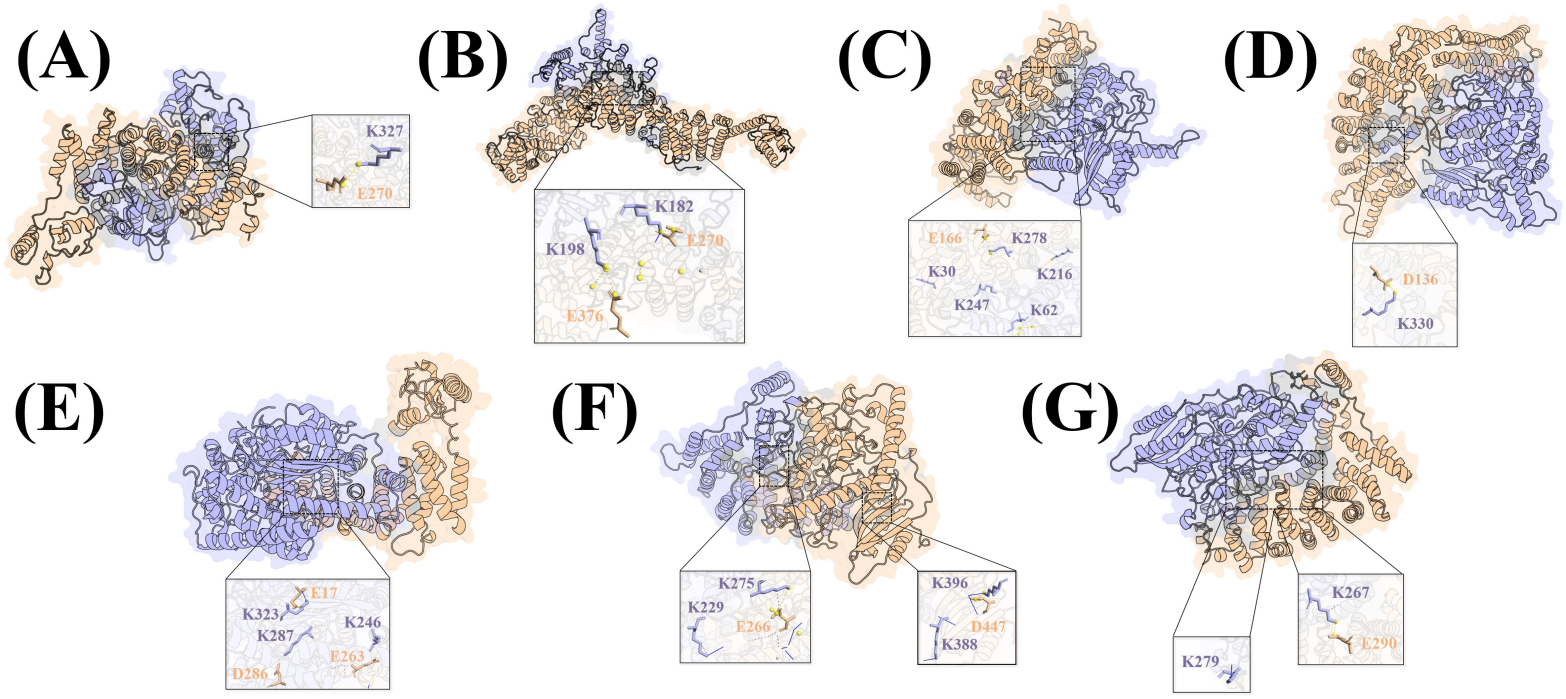
In all panels, Chain A (PUBs) is colored wheat, Chain B (ICE1s) light blue; core interaction interfaces are highlighted in light gray. **(A)** TpPUB24-TpICE1 complex; **(B)** SiPUB21-SiICE1 complex; **(C)** GmPUB23-GmICE1 complex; **(D)** VvPUB24-VvICE1 complex; **(E)** CaPUB1-CaICE1 complex; **(F)** MdPUB23-MdICE1 complex; **(G)** AtPUB26-AtICE1 complex.

**Figure 4:**
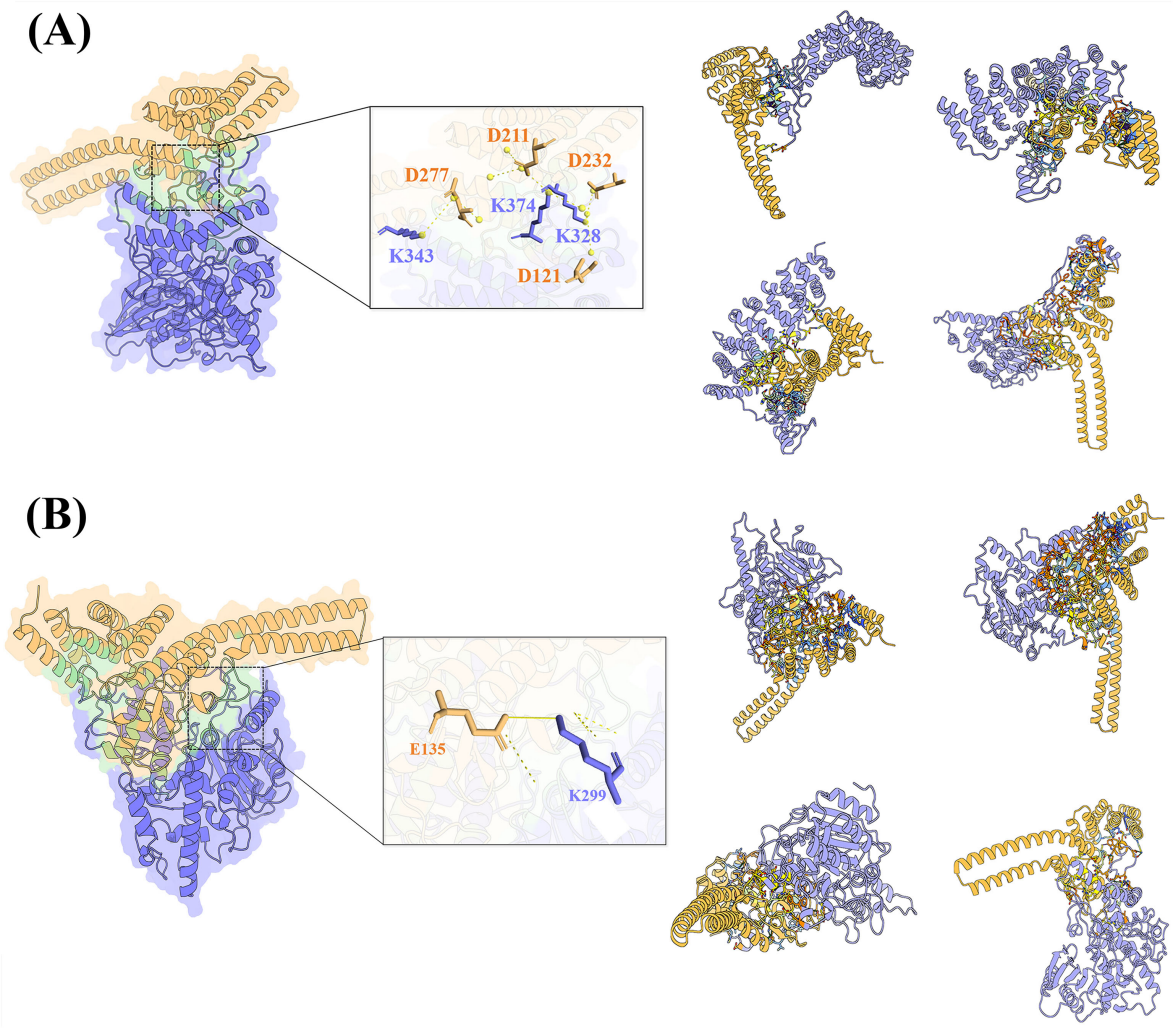
The AF2-predicted protein complex structures and interaction interface characterization. In all panels, Chain A (PUB protein) is colored light orange, Chain B (ICE1 protein) blue-purple; core interaction interfaces are highlighted in light green. **(A)** Structure of the AtICE1-MsPUB210 complex: Residues K343, K374 and K328 of AtICE1 interact with D277, D211 and D121 of MsPUB210, respectively. Right sub-panels display complex conformations colored by B-factor (pLDDT) scores. **(B)** Structure of the MsICE1-MsPUB210 complex: Residue E135 of MsICE1 interacts with K299 of MsPUB210. Right sub-panels display complex conformations colored by B-factor (pLDDT) scores.

**Figure 5:**
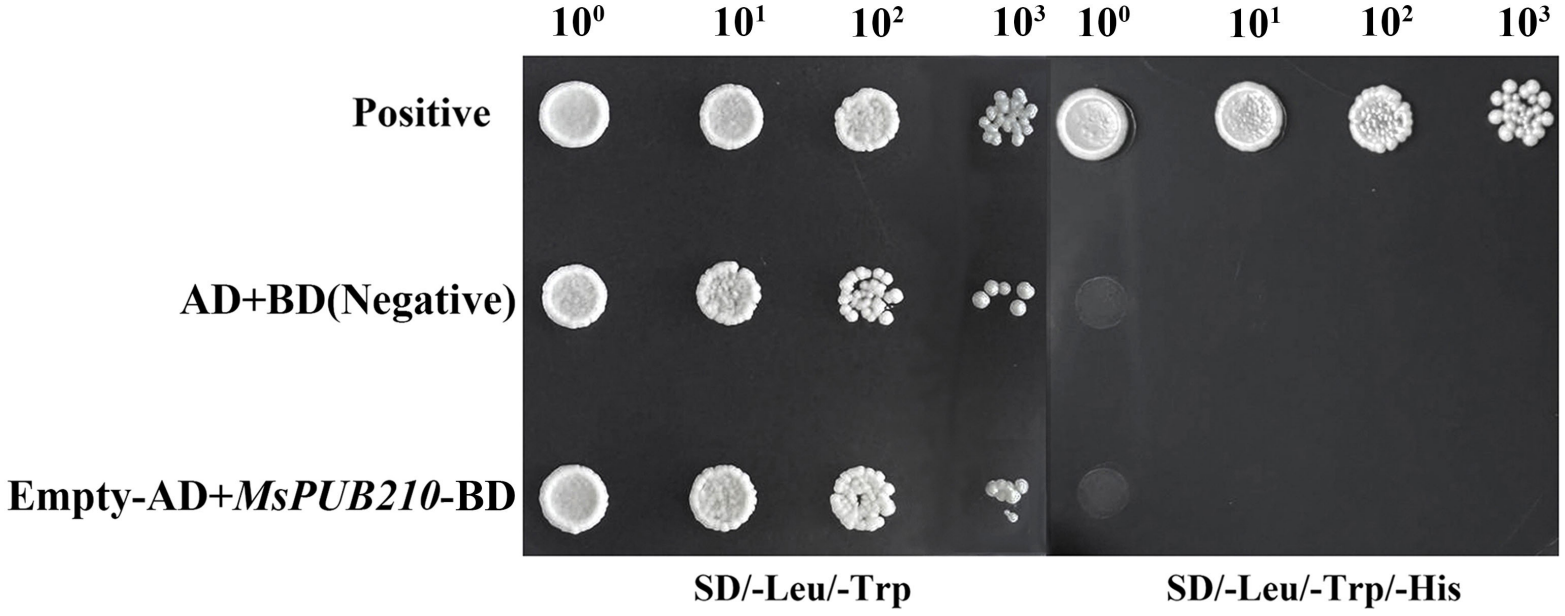
The transactivation validation experiment. The yeast transformants were grown on selective SD media supplemented contain 5 mM 3-AT with 3 independent experiments.

The phylogenetic tree revealed that MsPUB210 protein clusters closely with AtPUB53 protein, with a high bootstrap value of 98% (**Supplementary Figure 2**), and under the guidance of PPI network analysis and previous functional studies on the PUB gene family, we found that MsICE1 has a conserved domain, which has been proven to interact with PUBs (Yao et al., 2017; Wang et al., 2019; Li et al., 2024). Thus, we selected MsPUB210, MsICE1, and AtICE1 proteins for 3D complex structure prediction using AlphaFold2 (AF2)—this step aimed to provide structural support for the key regulatory links in the cold-responsive GRN. More AF2 models for other seven plant species (*T. pratense*, *S. italica*, *V. vinifera*, *C. annuum*, *M. domestica*, *A. thaliana*) were included to illustrate the broader conservation of potential interaction interfaces (**Figure 3**). The 3D structural models of the MsPUB210-MsICE1 and MsPUB210-AtICE1 heteromeric complexes along with their interfaces (including potential lysine ubiquitination sites), are presented in **Figure 4**. Consistent with evidence that later refinement cycles yield more stable protein structures (Jumper et al., 2021; Tang et al., 2022), we prioritized the structure from the final (5th) iterative refinement cycle for each complex across all seed-derived models. Two-dimensional (2D) diagrams of all interaction interfaces (containing K residues) are provided in **Supplementary Figures 3** and **4**. Following sequence verification, the full-length coding sequences of *MsPUB210* genes were cloned into the pGBKT7 (BD) yeast expression vector. All recombinants grew similarly on SD/-Trp/-His plates, while the positive control was the only one able to grow on SD/-Leu/-Trp/-His (5 mM 3-AT) plates, but the AD and BD domains with empty vectors, as well as the Empty-AD with *MsPUB210*-BD, all failed to exhibit growth. The observations indicated that *MsPUB210* does not exhibit intrinsic transcriptional activity in the yeast system (**Figure 5**). These results confirming the suitability of the experimental system for the subsequent two-hybrid assays.

### 3.3 MsPUB210 interacts with MsICE1 during cold stress to regulate its stability

**Figure 6:**
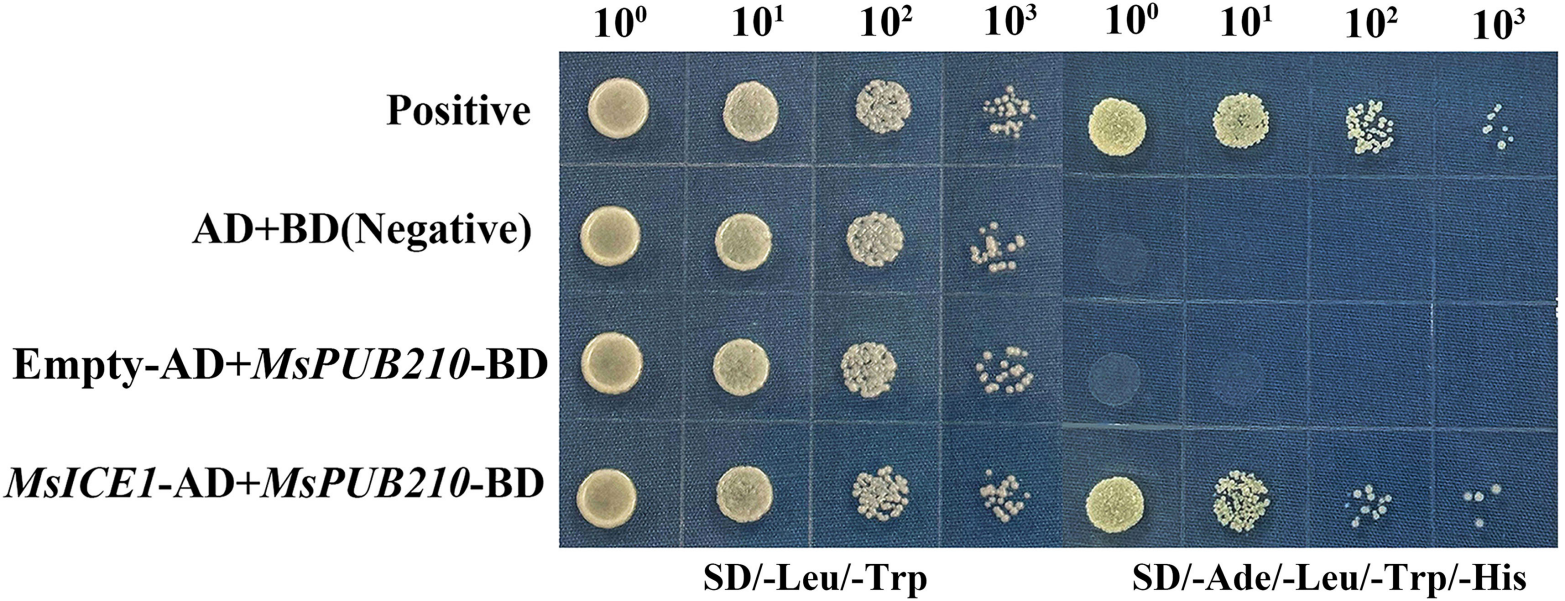
MsPUB210 was shown to interact with the MsICE1 in yeast two-hybrid (Y2H) assay. Yeast colonies were grown on selective SD media contains 5 mM 3-AT. Similar results came from 3 independent experiments.

Combined with GRN-oriented PPI prediction results and AF2 structural evidence, we conducted yeast two-hybrid (Y2H) assays to verify whether MsPUB210 physically interacts with MsICE1—this interaction is a critical basis for confirming the regulatory relationship between the two genes in the cold-responsive GRN. The Y2H system was validated in previous transactivation activity assay. The full lengthen of *MsPUB210* was fused to the BD and *MsICE1* was fused to the AD. After the co-transformed into yeast, we observed strong and specific reporter activation only in the yeast cells which carrying both *MsPUB210*-BD and *MsICE1*-AD constructs (**Figure 6**). This indicates a direct interaction between *MsPUB210* and *MsICE1* in yeast, also consistent with the PPI prediction.

### 3.4 Isolation, cloning and sequence analysis of the *MsPUB210* gene

The CDS region of *MsPUB210* was amplified via PCR using *Medicago sativa* cDNA as the template and gene-specific primers. The PCR amplicons were subjected to agarose gel electrophoresis for validation, with the corresponding results in **Supplementary Figure 5**. We obtained the full open reading frame (ORF) of *MsPUB210* from NCBI. It spans 783 bp, encoding a polypeptide of 277 amino acids. Based on ProParam predictions, the encoded protein has a molecular formula of C_1415_H_2212_N_404_O_424_S_10_, a molecular weight of 31.99 kDa, and a theoretical isoelectric (pI) of 6.08. With an instability index calculated as 41.80, MsPUB210 was predicted to be an unstable and acidic protein. Composed of all 20 types of amino acids, the protein is rich in Ala (9.0%), Arg (6.5%), and Asn (4.7%), with 38 positively charged and 44 negatively charged residues in total. The aliphatic index is 80.00, the predicted half-life is 30 h and the grand average of hydropathicity (GRAVY) is -0.651. WoLF PSORT was used to assess the subcellular localization of MsPUB210 to be primarily in the nucleus with low potentially localized in the cytoplasm, plasma membrane, chloroplast, and mitochondrion at low levels.

### 3.5 Functional validation of *MsPUB210* in enhancing cold stress tolerance of transgenic Arabidopsis

**Figure 7:**
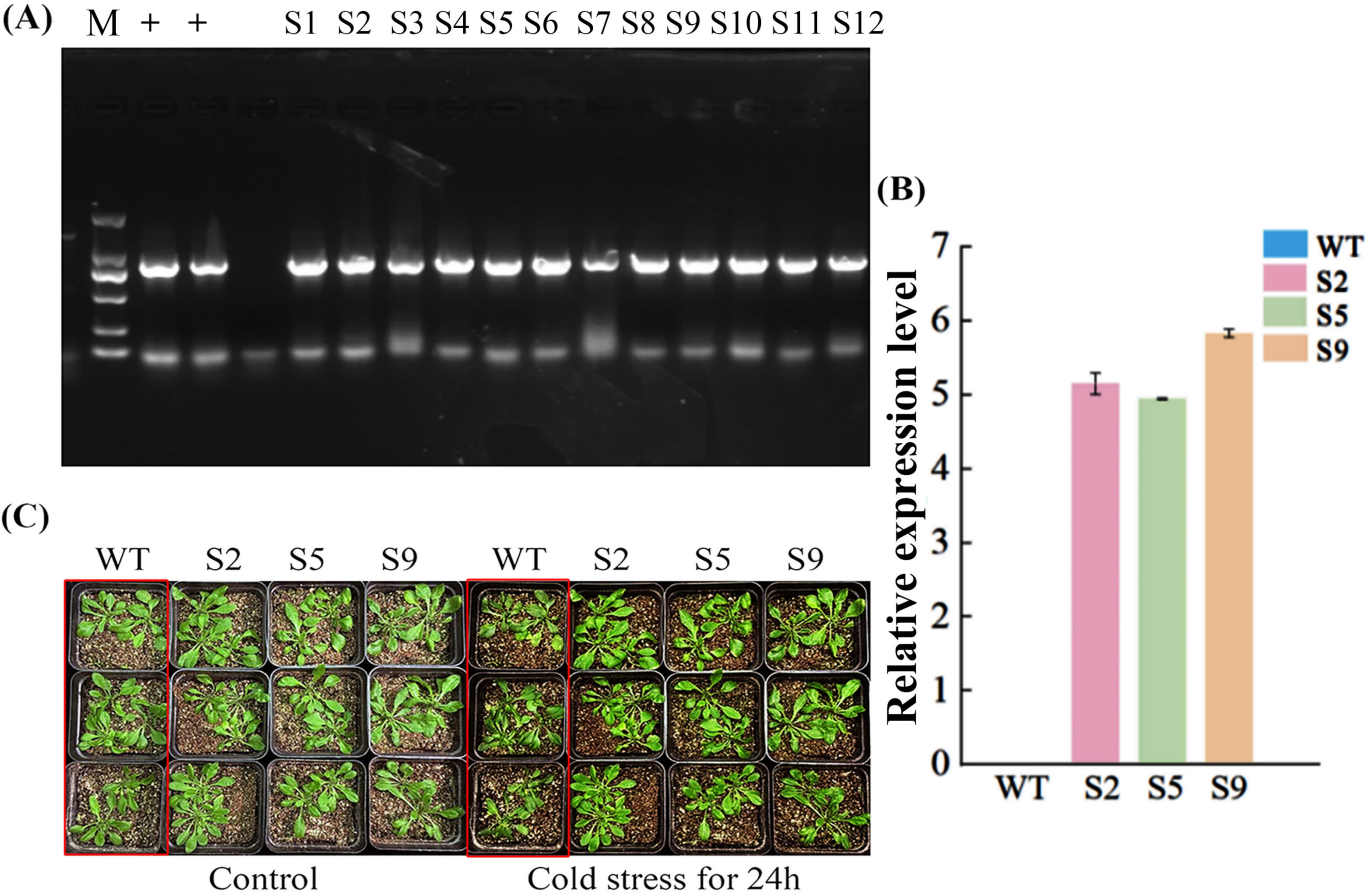
Identification of transgenic MsPUB210 Arabidopsis thaliana and phenotypic map under cold stress. All assays were performed with three biological replicates and three technical replicates, and data are presented as mean ± standard deviation (SD). **(A)** The transgenic MsPUB210 Arabidopsis thaliana was identified with 2000 bp marker and validated with negative (-), positive (+) controls. A total of 12 strains were identified as S1~S12. **(B)** Successful expression of MsPUB210 in homozygous transgenic lines (S2, S5, S9), no expression in WT plants. **(C)** Phenotype plots of WT and transgenic Arabidopsis after control and cold stress treatments.

To investigate the *in vivo* biological functions of *MsPUB210*, we conducted a series of heterologous overexpression assays in *Arabidopsis thaliana*. We constructed the pCAMBIA1300-*MsPUB210* strand, then transferred it into *Agrobacterium tumefaciens* and infected Arabidopsis inflorescences via the floral-dip method. After selection on kanamycin-containing medium, multiple transgenic lines were obtained. The transgenic lines were verified by genomic PCR with specific primers which revealed that the *MsPUB210*-specific fragments showed clear amplification bands while WT had no detectable amplification (**Figure 7A**). We selected the homozygous lines S2, S5, and S9 of these transgenic plants in successive generations for the following quantified analysis. Extract the total RNAs from the lines above, then reverse-transcribe into cDNA for qRT-PCR. Real-time PCR quantified *MsPUB210* in transgenic lines (**Figure 7B**), revealing a 5-6-fold increase in relative expression levels compared to WT. The observed differences between the WT and lines S2, S5 and S9 enabled further investigation of the mechanism by which *MsPUB210* regulates cold stress tolerance. Cold stress was applied to four-week-old seedlings at 4°C for 24 hours. Visually, WT plants showed severe cold damage after stress: leaves exhibited obvious wilting, edge curling, and irreversible shrinkage due to water loss. In contrast, *MsPUB210*-overexpressing lines (S2, S5, S9) only showed slight leaf wrinkling, retained bright green color and stretched morphology (WT damage phenotypes are highlighted in the figure for clarity), which directly confirming the physiological advantages of transgenic lines (**Figure 7C**).

### 3.6 Expression profiling of resistance-related genes in *MsPUB210*-transgenic Arabidopsis under low temperature

**Figure 8:**
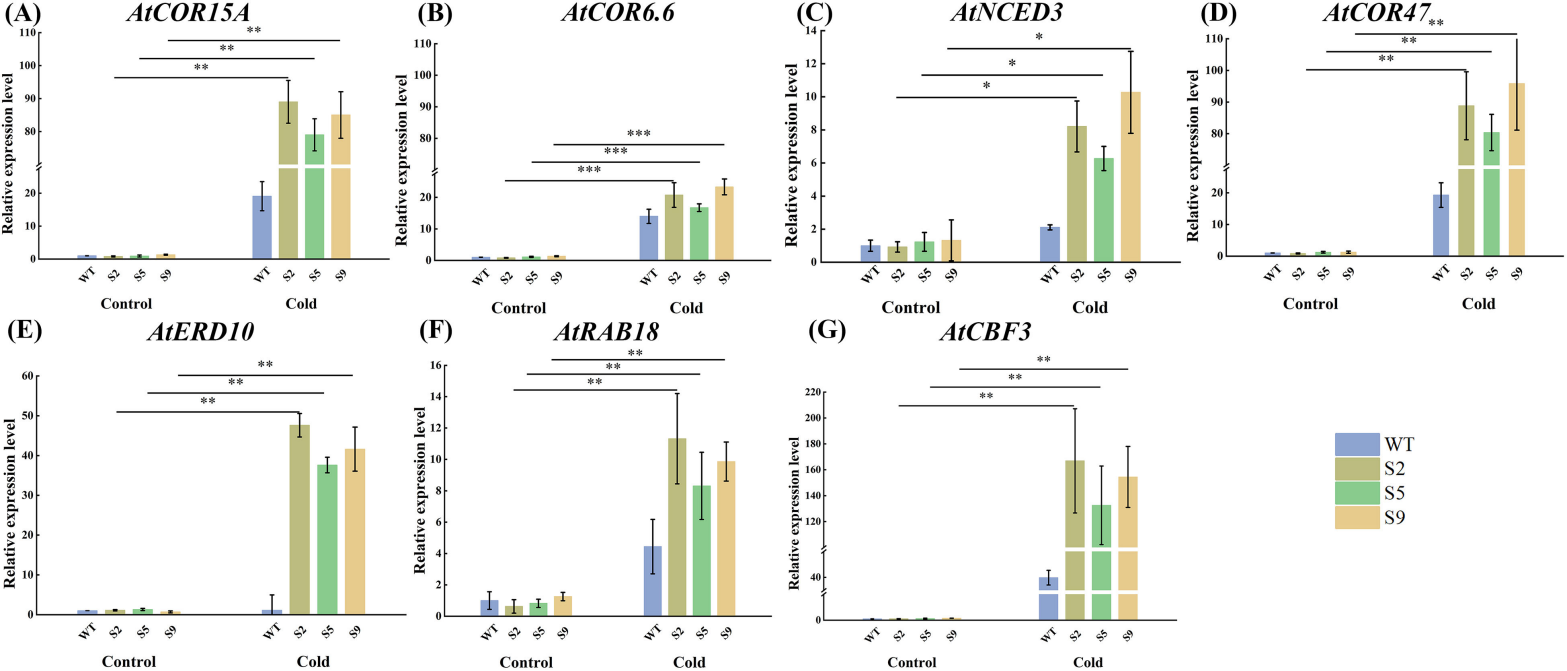
Expression of cold-related genes in *Arabidopsis thaliana* transgenic for *MsPUB210* under low-temperature stress. **(A)** *AtCOR15A*; **(B)** *AtCOR6.6*; **(C)** *AtNCED3*; **(D)** *AtCOR47*; **(E)** *AtERD10*; **(F)** *AtRAB18*; **(G)** *AtCBF3*. The x-axis represents the control and treatment groups; the y-axis represents the relative expression levels of the genes. Different colors represent WT, S2, S5 and S9 separately. The expression level of control WT was 1. The relative expression level was calculated using the 2^-△△CT^ method formula. All assays were performed with three biological replicates and three technical replicates, and data are presented as mean ± standard deviation (SD). Statistical significance was determined via the one-way analysis of variance (one-way ANOVA) with *P* < 0.001 marked as ***, *P* < 0.01 marked as **, *P* < 0.05 marked as *.

To dissect the transcriptional regulatory role of *MsPUB210* in cold stress responses, we selected three independent *MsPUB210*-overexpressing Arabidopsis lines (S2, S5, S9) and wild-type (WT) plants, exposing them to 4 °C cold treatment for 24 h (with three biological, three technical replicates per group). We then quantified the expression of canonical cold-responsive marker genes by qRT-PCR. Gene expression levels were normalized to the reference gene *GAPDH*. We targeted 7 key cold-responsive genes for quantification, each with well-characterized functions in plant cold signaling based on previous studies and PPI networks. As shown in **Figure 8**, the expression levels of these genes were significantly higher in *MsPUB210*-transgenic lines that WT plants following cold treatment. *AtCBF3*, *AtCOR15A*, *AtCOR47* and *AtCOR6.6* were highly expressed in each line. When comparing each line’s cold-stressed subgroup to its own non-stressed counterpart, cold-responsive genes were upregulated in both WT and overexpressing lines after stress exposure. Critically, the upregulation magnitude of these genes in cold-stressed overexpressing lines was significantly greater than that in the cold-stressed WT group.

### 3.7 *MsPUB210* improves physiological adaptation to cold stress in transgenic Arabidopsis

**Figure 9:**
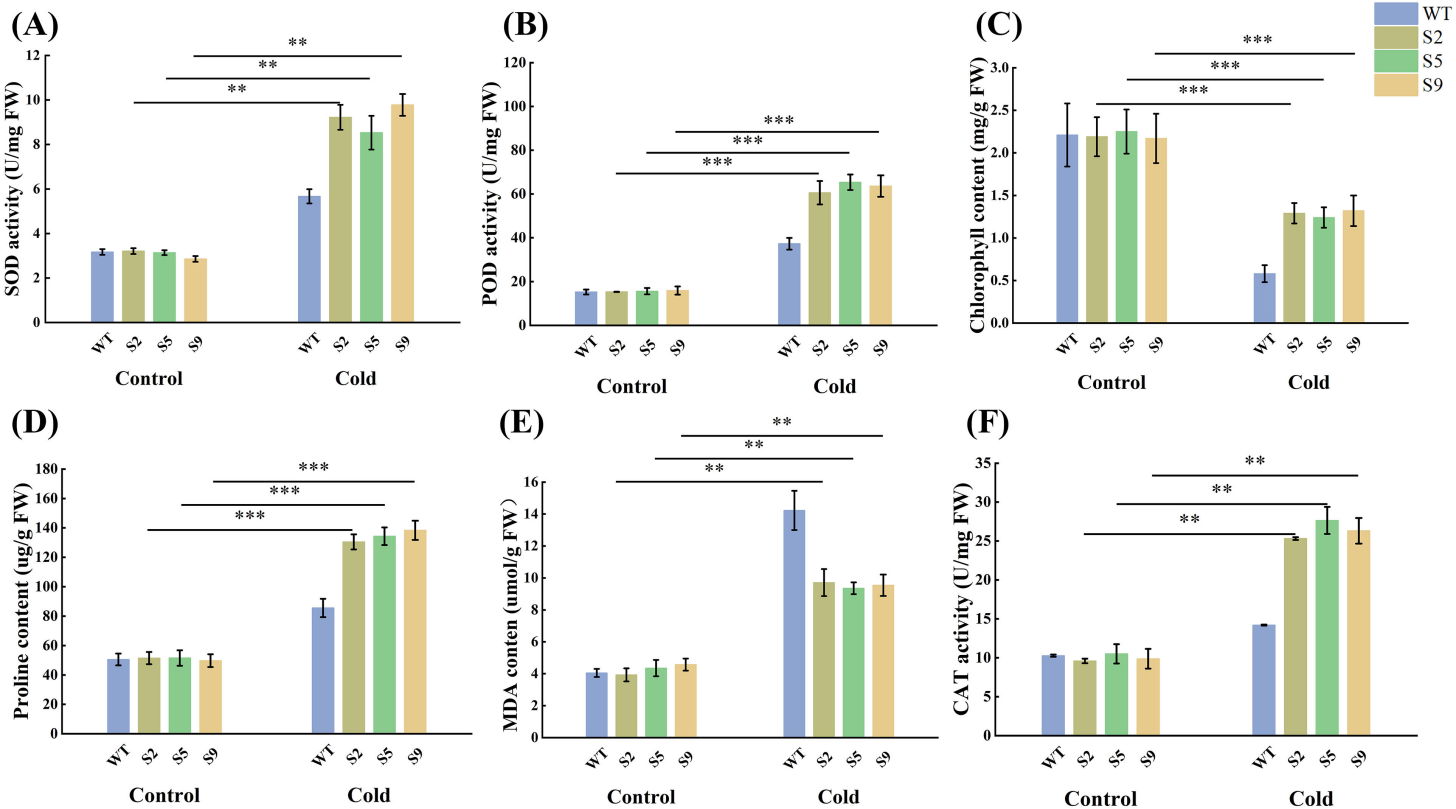
**(A)** SOD content; **(B)** POD activity; **(C)** CHL content; **(D)** Proline content; **(E)** MDA content; **(F)** CAT activity. The x-axis represents the control and treatment groups; the y-axis represents the activity or content. All assays were performed with three biological replicates and three technical replicates, and data are presented as mean ± standard deviation (SD). Different colors represent WT, S2, S5 and S9 separately. Statistical significance was determined via the one-way analysis of variance (one-way ANOVA) with *P* < 0.001 marked as ***, *P* < 0.01 marked as **, *P* < 0.05 marked as *.

The effect of *MsPUB210* on physiological responses and whether it improves *Arabidopsis thaliana*’s cold tolerance was validated. After low-temperature treatment, physiological parameters of transgenic lines and WT plants were quantified and compared with those under normal conditions We found all transgenic lines performed better than WT. The level of chlorophyll remained stably higher in lines than in WT, suggesting a reduced rate of chlorophyll degradation. Although the less decline of chlorophyll could indicate improved photosynthetic efficiency, more convincing biophysical analyses, such as PAM or chlorophyll fluorescence, are required for further validation. Furthermore, the transgenic plants accumulate less MDA than wild-type plants, indicating less lipid peroxidation and improved membrane integrity. Proline accumulation significantly increased in transgenic lines compared with the WT, showing better osmoregulatory properties compared to WT plants. In addition, the activities of major antioxidant enzymes, including CAT, POD and SOD were also higher in transgenic plants. These quantitative differences were consistently observed across independent transgenic lines (**Figure 9**).

## 4 Discussion

In plants, U-box E3 ligases have been widely recognized as regulators of stress responses through diverse mechanisms, with members identified in many crop species, including rice, grapevine, apple, maize and soybean (Wang et al., 2016; Wang et al., 2022; Yao et al., 2017; Yoo et al., 2020; Wang et al., 2023a), suggesting a broadly conserved role in abiotic stress adaptation across diverse plant taxa (Osborne, 2024). In the model plant *Arabidopsis thaliana*, PUB25 and PUB26 have been shown to interact directly with ICE1, a central regulator of cold signaling, to modulate its stability via distinct ubiquitin chain topologies at different stages of cold exposure (Wang et al., 2023b). These studies established that U-box E3 ligases can fine-tune CBF expression and cold tolerance by controlling key transcriptional regulators, a dynamic regulatory pattern that shapes downstream cold adaptation pathways. Notably, this PUB-ICE1 regulatory module is not unique to Arabidopsis. The conserved U-box domain enables these proteins to recognize specific substrates and mediate their ubiquitination, and similar roles have been reported in other species, such as pepper and cucumber, where U-box genes participate in cold stress responses through conserved domain functions (Min et al., 2016; Chen et al., 2025). Unlike these conventional U-box ligases, PQT3 belongs to a unique *Arabidopsis* RING-finger/U-box E3 ligase family. Its core biological function is to terminate the activated oxidative stress response by targeting PRMT4b—a histone-modifying protein—for ubiquitin-dependent degradation, altering the expression of critical antioxidant enzymes including APX1 and GPX1. This example illustrates how ubiquitination modulates overlapping stress signaling networks (e.g., ROS pathways triggered by cold stress), expanding the scope of U-box-mediated stress regulation beyond the CBF-ICE module (Luo et al., 2016).

In *Medicago sativa*, gene family analysis identified 210 U-box genes, indicating a complex and potentially specialized set of regulators for environmental response (Li et al., 2024). Among these, *MsPUB210* was predicted to possess E3 ligase activity and to target ICE1-like proteins based on sequence homology and domain structure.

ICE1 is a well-characterized MYC-type bHLH transcription factor and a core positive regulator in the conserved ICE-CBF-COR cold signaling pathway across plant species (Chinnusamy et al., 2003). For example, its apple ortholog MdCIbHLH1 also harbors a conserved bHLH domain and enhances cold tolerance by regulating *CBF* expression (Feng et al., 2012). In *Arabidopsis thaliana*, additional bHLH proteins MYC70 interact with ICE1 to fine-tune *CBF* expression and cold tolerance, further underscoring the functional significance of bHLH protein interactions in this pathway (Ohta et al., 2018). Cold signaling in plants is strongly linked to this cascade, and Dong et al. first demonstrated that overexpressing *ICE1* increases *CBF* expression, laying a foundation for subsequent studies (Dong et al., 2006). Moreover, ICE1 interacts with various regulatory factors to modulate downstream gene expression in multiple species: *MsLHY* upregulates *MsICE1*, *MsCBF1*, and *MsCOR15A* in *Medicago sativa* to enhance chilling stress defense (Li et al., 2025), while SIZ1 stabilizes ICE1 to facilitate the expression of *CBF3/DREB1A*, thereby enhancing cold tolerance (Zhang et al., 2016).

A key methodological consideration in this study is the use of PPI network analysis as a tool to infer and dissect the cold-responsive gene regulatory network (GRN). To elucidate the molecular mechanism underlying the enhanced cold-tolerance phenotype, we focused our analysis on GRN-oriented PPI networks and AlphaFold-Multimer (AF2-Multimer) predictions, combined with rigorous experimental validation. The GRN first provided preliminary clues for the interaction between PUB proteins and ICE1—a regulatory relationship previously characterized in *A. thaliana* (Cyr et al., 2002; Cho et al., 2008) and further supported by recent cold signaling GRN studies (Li et al., 2024). Based on their studies, our PPI analysis retrieval showed that PUB-ICE1 gene pairs across diverse plant species all exhibit interactions in the STRING database. AF2-Multimer predictions generated high-confidence structural models of the MsPUB210-MsICE1/AtICE1 complexes, and the consistency between the predicted interfaces and the robust Y2H positive signals directly validated the reliability of the regulatory link between these two core nodes in the GRN.

To further validate the broad conservation of the PUB-ICE1 interactions, we extended our AF2-Multimer analyses to PUB-ICE1 protein pairs across 6 plant species, in addition to the native MsPUB210*-*MsICE1 complex from *Medicago sativa*; notably, our native *MsPUB210-MsICE1* prediction also showed strong consistency with robust positive signals from yeast two-hybrid (Y2H) assays, confirming that the predicted protein interfaces mediate biologically relevant physical binding. Olsvik et al. emphasized the power of integrating structural modeling with phylogenetic analysis to identify functional motifs and domains, a strategy that further supports the reliability of our AF2-based interaction predictions (Olsvik and Johansen, 2023). Moreover, this integrated computational-experimental workflow—combining AlphaFold-guided PPI prediction with subsequent experimental validation—aligns with established methodologies in protein interaction studies: for instance, Abulude et al. corroborated the predicted Bd0075-Bd0474 interaction via the BACTH system (Abulude et al., 2025), while Lee et al. demonstrated that AF2-Multimer serves as a core predictor in high-throughput PPI screening pipelines, with top candidates validated via luminescence, fluorescence assays, or Y2H (Lee et al., 2024). The AF2-predicted structures of the MsPUB210-MsICE1 complex exhibit structural flexibility an essential property for dynamic PPI as highlighted in both structural studies (Yang et al., 2021). Collectively, these studies validate the robustness of our approach. When combined with the results of yeast transactivation assays (a critical control to rule out false positives in Y2H experiments), our AF2-Multimer and Y2H data lay a solid foundation for subsequent mechanistic discussions.

Yeast two-hybrid (Y2H) assays further confirmed a direct physical interaction between MsPUB210 and MsICE1, consistent with our AF2-Multimer predictions. To delineate the molecular mechanism by which *MsPUB210* regulates cold tolerance, we performed expression analyses of 7 key cold-responsive genes. Combining these expression data with the inherent function of PUB proteins as U-box E3 ubiquitin ligases, we propose the following regulatory mechanism: under low-temperature conditions, MsPUB210 targets two cores cold signaling regulators—ICE1 and DREB—for ubiquitination. This post-translational modification directly upregulates the expression of downstream CBF transcription factors, thereby triggering a cascading activation of COR and ERD genes in the cold-signaling pathway and ultimately enhancing plant cold tolerance. We hypothesize that this interaction is mediated by the conserved bHLH domain of MsICE1 protein or its associated protein interfaces, suggesting that MsPUB210 protein engages the cold signaling cascade by recognizing conserved structural motifs shared by ICE1 and its related bHLH family members.

In this study, we combined AlphaFold2 (AF2) structural prediction with PPI analysis: the high-confidence 3D structures of the MsPUB210-ICE1 complexes predicted by AF2 not only confirmed the feasibility of the protein interactions inferred from PPI but also provided structural evidence for the stability of the regulatory link between these two core nodes in the GRN. Subsequent Y2H experiments further validated the core interaction, forming a complete evidence chain of “GRN inference via PPI & structural corroboration via AF2 & experimental validation via Y2H”. Notably, the downstream cold-responsive genes (e.g., *CBF*s, *COR*s, *NCED*s) linked to MsPUB210 and ICE1 in the PPI network are well-characterized core nodes in the plant cold-responsive GRN—this consistency further confirms that our PPI-based approach effectively captures key regulatory modules of the cold-responsive GRN.

To further confirm the biological relevance of the MsPUB210-ICE1 interaction and dissect the functional role of *MsPUB210* in cold stress response, we first performed a phylogenetic analysis of PUB genes across diverse plant species. This analysis identified homologous PUB proteins, revealing that AtPUB22 and AtPUB23 are closely related to CaPUB1, which also shares 52%–60% similarity with orthologs from tobacco, tomato, and parsley (Min et al., 2016). Importantly, our study verified the homology between MsPUB210 and AtPUB53, providing a solid theoretical basis for the plausibility of heterologous overexpression of *MsPUB210* in *Arabidopsis thaliana*. The network exhibits high pathway clarity, distinctly resolving a core interaction module anchored by PUB-ICE1, with sub-networks linking to downstream cold stress effectors.

This molecular regulation presumably mediated by the conserved structural motifs of ICE1 and its homologous, directly translates to enhanced physiological performance in *MsPUB210* overexpression lines: transgenic lines exhibited higher survival rates, improved photosynthetic efficiency, and elevated activities of key cold-responsive enzymes, alongside stronger induction of canonical cold marker genes (e.g., *CBF*s, *COR*s, *ERD*s). Specifically, the upregulation of *AtCBF3* (a core ICE-CBF-COR cascade transcription factor) aligns with our prior GRN predictions (Section 3.1); *AtCOR15A* and *AtCOR47*’s elevated transcription corresponds to the lower MDA accumulation and improved cellular stability observed in Section 3.7; and *AtCOR6.6*’s induction supports the protection of photosystem II from cold-induced photoinhibition. The better activity of core components of the plant antioxidant system such as superoxide dismutase (SOD), peroxidase (POD), and catalase (CAT) also indicates that the antioxidant defense capacity was enhanced in transgenic plants. SOD eliminates toxic superoxide anion, while POD and CAT decompose hydrogen peroxide (H_2_O_2_) into harmless water and oxygen. These molecular and physiological observations further confirm that *MsPUB210* enhances cellular stability and mitigates oxidative damage during cold stress.

Interestingly, the upregulation of ABA-related genes such as *RAB* and *NCED* in *MsPUB210* transgenic lines suggests the involvement of ABA signaling in the cold response regulated by PUB ligases. Kelley et al. concluded that several ubiquitin-mediated controls of plant signaling exist, and that inducing PUBs may contribute to ABA signaling (Kelley and Estelle, 2012). Previous work has indicated that ubiquitin-mediated mechanisms may intersect with ABA pathways during stress responses, implying that PUBs contribute to multiple regulatory networks beyond direct CBF activation (Su et al., 2024). More recently, Chang and Chung reinforced this intersection via in silico AlphaFold2 structural analyses: their models predicted conserved interaction interfaces between E3 ligases and ABA receptors, providing structural support for the hypothesis that PUBs physically engage ABA signaling components to modulate their function (Chung et al., 2025; Du and Zhang, 2025), which is supported by our findings.

**Figure 10:**
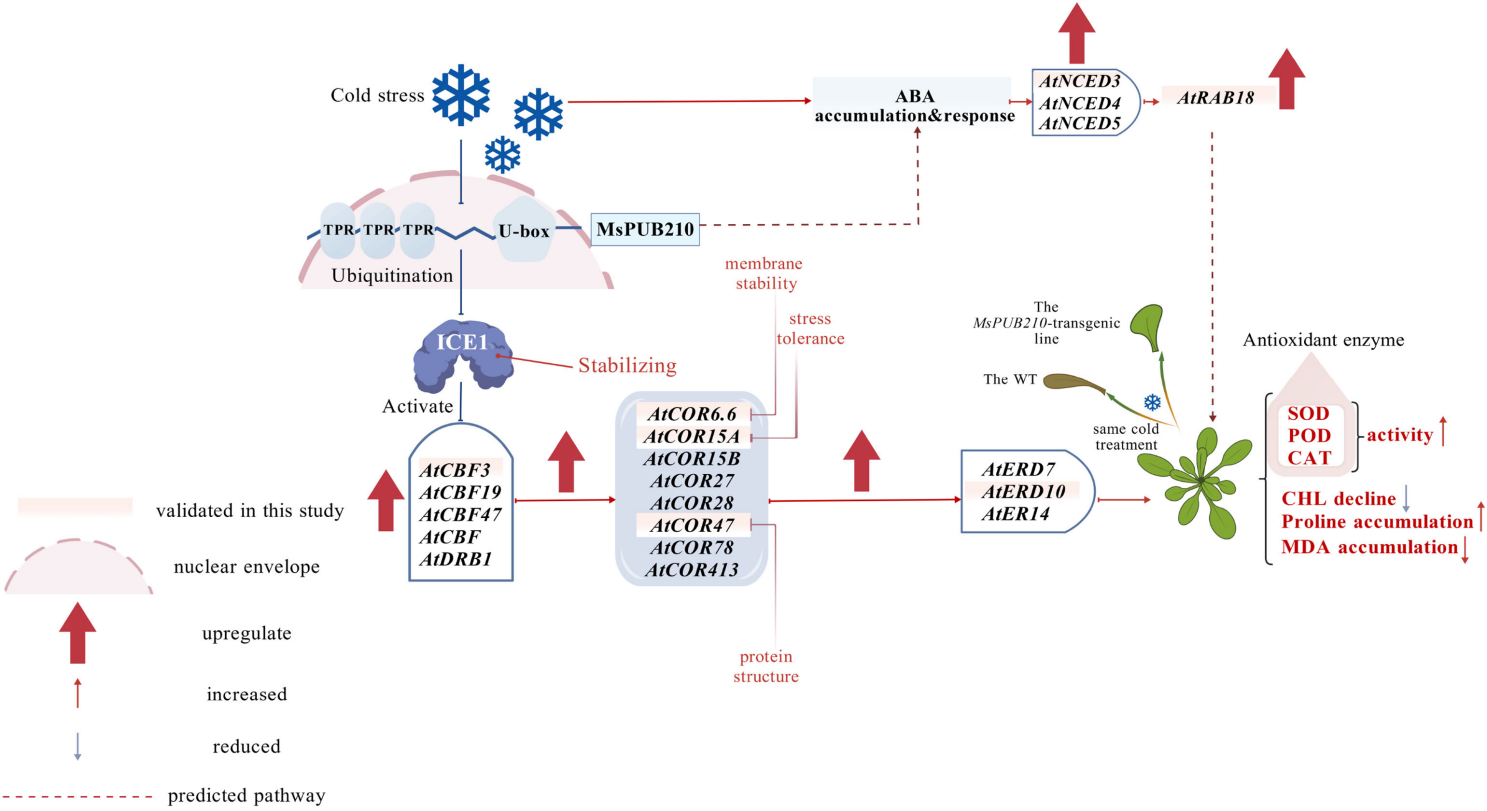
The proposed mechanism by which *MsPUB210* regulates plant cold tolerance. Under chilling stress (4 °C), the U-box E3 ligase gene *MsPUB210* in *Medicago sativa L.* is upregulated. The MsPUB210 protein interacts with the ICE1 protein via its conserved U-box domain and mediates ICE1 ubiquitination (supported by in silico prediction of lysine (K) ubiquitination sites at the interaction interface). This ubiquitination event likely stabilizes ICE1 or enhances its transcriptional activity, thereby activating the downstream conserved ICE-CBF-COR pathway: ICE1 directly upregulates the expression of *CBF* family genes, which in turn induces the transcription of cold-responsive genes. Concurrently, *MsPUB210* coordinates crosstalk with the ABA signaling pathway by promoting the upregulation of ABA-related genes, further reinforcing cold stress adaptation. Collectively, these molecular events enhance plant chilling tolerance through multiple physiological improvements: Solid lines indicate validated regulatory links (e.g., Y2H-verified PPI, qRT-PCR-confirmed gene upregulation), while dashed lines represent predicted pathways (e.g., ABA accumulation).

Taken together, our findings clarify the specific role of *MsPUB210* in *Medicago sativa* cold adaptation and complete the previously unidentified specific function of this gene. We demonstrate that PUBs function as the positive regulators of cold tolerance by interacting with the conserved sequence of ICE1, modulating downstream *CBF* genes, and activating the ICE-CBF-COR pathway (**Figure 10**). Our work not only elucidates the molecular mechanism by which PUBs improve the cold tolerance of *Medicago sativa* but also provides new insights into the functional diversity of PUB E3 ubiquitin ligases in plant cold stress responses. The mechanism diagram was drawn using Bio-DGP (Jiang et al., 2025).

This study’s limitations result in negative validation, such as loss-of-function experiments (gene knockout or knockdown) using CRISPR-Cas9 or RNAi to examine the necessity of *MsPUB210*, which strongly confirmed *MsPUB210*’s positive regulation of the cold response. More pull-down assays will be performed to confirm *MsPUB210*’s *in vitro* activity and ensure that the Y2H results are convincing. While direct biochemical evidence of MsPUB210-mediated ICE1 ubiquitination awaits follow-up validation, our AF2-predicted ubiquitination sites at the interaction interface (Section 3.2), Y2H-verified MsPUB210-MsICE1 interaction (Section 3.3), and conserved PUB-ICE1 ubiquitination mechanisms in plants collectively support this regulatory link; subsequent *in vitro*/*in vivo* ubiquitination assays will further confirm this cascade. Future studies will focus on *Medicago sativa*, conducting research with the hairy root system to characterize *MsPUB210* (Nawy et al., 2005); determine whether it targets other repressors in the cold signaling pathway.

## 5 Conclusions

Despite considerable progress in plant U-box E3 ubiquitin ligase (PUB) research, the functional characterization of many PUB family members and their precise regulatory mechanisms in abiotic stress responses remain largely elusive. In this study, we used an integrated approach combining GRN-oriented PPI network analysis, AlphaFold2 (AF2) structural prediction, and experimental validation to provide compelling evidence for a conserved cold adaptation mechanism in plants: the U-box gene *MsPUB210*, a novel regulatory node in the cold-responsive GRN, mediates the ubiquitination of the key cold signaling regulator ICE1. This post-translational modification of ICE1 orchestrates a cascade of physiological and molecular responses that collectively enhance plant cold tolerance, including reduced membrane lipid peroxidation (improved membrane stability), strengthened antioxidant defense systems via elevated antioxidant enzyme activities and robust activation of downstream cold-responsive marker genes in the GRN (e.g., *AtCBF*, *AtCOR*, *AtERD10*, *AtRAB18*, and *AtNCED3*). Furthermore, yeast two-hybrid (Y2H) assays confirmed a direct physical interaction between MsPUB210 and MsICE1—a key basis for their regulatory relationship in the GRN—which was further supported by high-confidence AF2-predicted 3D structures of the complexes. This finding not only identifies *MsPUB210* as a novel regulatory component in the conserved ICE-CBF-COR cold-responsive GRN but also provides a new target for functional mining of cold-tolerant genes in the plant U-box family. Collectively, our work lays a solid foundation for further exploring the diverse roles of U-box genes in the plant abiotic stress-responsive GRNs and offers a valuable theoretical basis and technical reference for the genetic improvement of alfalfa (*Medicago sativa L.*) to enhance its cold tolerance and ultimately increase crop yields.

## Data Availability

The original contributions presented in the study are included in the article/**Supplementary Material**. Further inquiries can be directed to the corresponding author.
