# Supplementary material for: The alfalfa U-box E3 ligase MsPUB210 interacts with MsICE1 and positively regulates cold tolerance in transgenic Arabidopsis
Source: Front Plant Sci. 2026 Mar 13;17:1772992. doi: 10.3389/fpls.2026.1772992 (PMC13021411; doi:10.3389/fpls.2026.1772992)
Supplement: Supplementary file 1 [file Supplementaryfile1.docx]

Supplementary Table 1. The pGKBT7 and pGADT7 primers for MsPUB210-BD and MsICE1-AD construct

| Primer Name | Primer Sequence (5’-3’) |
| --- | --- |
| *MsPUB210BD*-F | CATGGAGGCCGAATTCATAGTGAAATGAGGCCAAAC |
| *MsPUB210BD*-R | GGATCCCCGGGAATTCAAACCTCTCACAAACTGCTA |
| *MsICE1AD*-F | GGAGGCCAGTGAATTCTTCTCTCTCTCCTTAAACAAACCCA |
| *MsICE1AD*-R | CACCCGGGTGGAATTCTTAAACTGAAGTCACCAGAGCCAAT |

Supplementary Table 2. Primers for MsPUB210, MsICE1 cloning and vector linkage.

| Primer Name | Primer Sequence (5’-3’) |
| --- | --- |
| *MsPUB210*-F | ATAGTGAAATGAGGCCAAAC |
| *MsPUB210*-R | AAACCTCTCACAAACTGCTA |
| *MsICE1*-F | TTCTCTCTCTCCTTAAACAAACCCA |
| *MsICE1*-R | TTAAACTGAAGTCACCAGAGCCAAT |


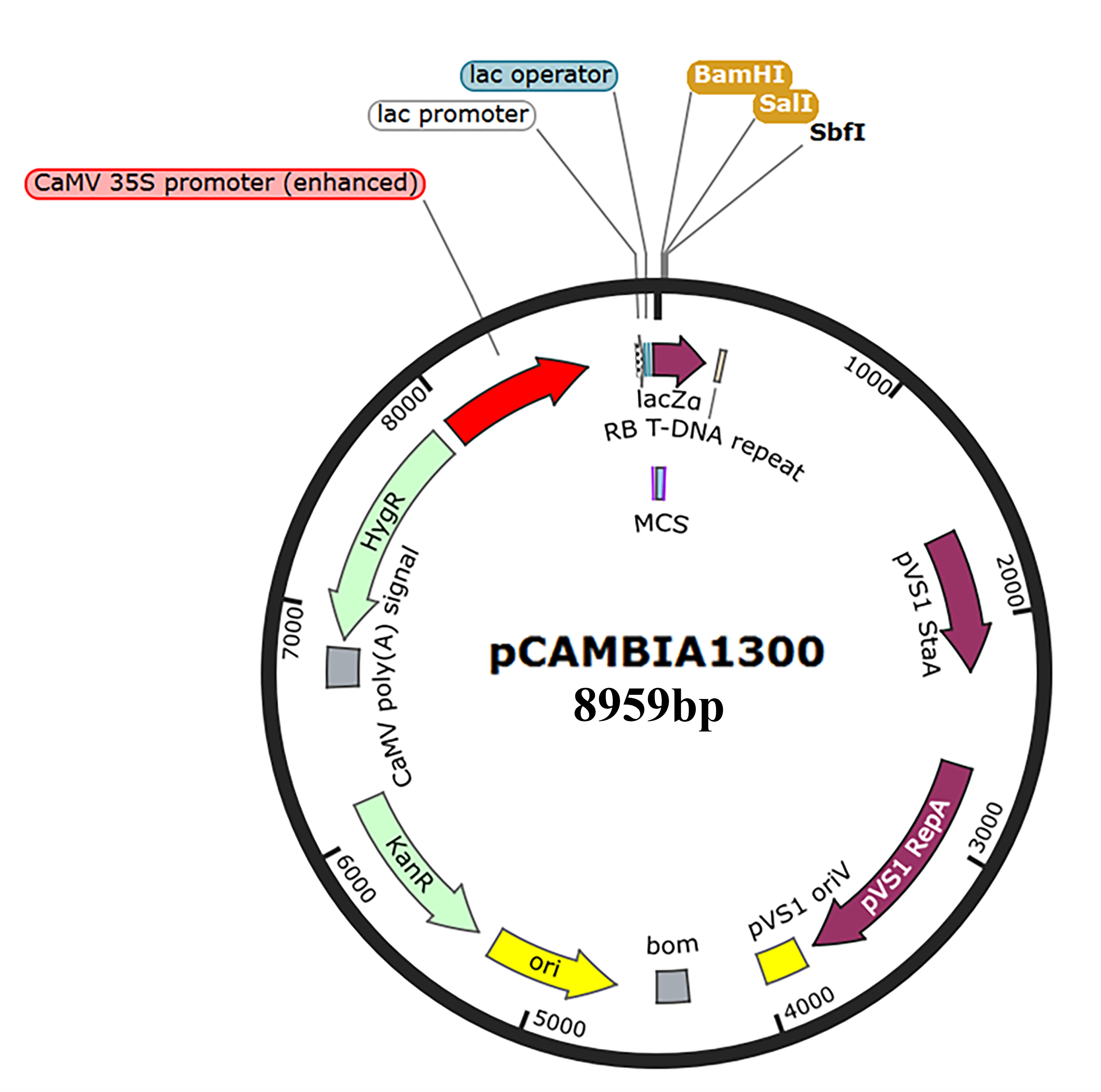


Supplementary Figure 1.Map of pCAMBIA1300 vector (8959 bp in full length). The red-labeled enhanced CaMV 35S promoter as the default promoter, the restriction enzyme sites (BamHI, SalI) was orange-labeled.

Supplementary Table 3. Primers of *MsPUB210* overexpression validation

analysis

| Primer Name | F | R |
| --- | --- | --- |
| *MsPUB210* | CAGTTGGAGGCTGTAGAAAG | TTCATGGACTGCTTCCCTAA |

Supplementary Table 4. Specific primers for downstream gene qRT-PCR analysis

| Primer Name | Primer Sequence (5’-3’) |
| --- | --- |
| *AtRAB18*-F | GTCAGGACAACCCGAATTT |
| *AtRAB18*-R | ATACGAACTTGTTAGCGTCC |
| *AtNCED3*-F | GGTGGTTTACGACAAGAACA |
| *AtNCED3*-R | GAGTCTGGTGGAGTCATACA |
| *AtERD10*-F | ACATTCGGTAGAGGATCACA |
| *AtERD10*-R | TTCCTCTCCAGTGGTCTTG |
| *AtCBF3*-F | AGGCGGTGATTATATTCCGA |
| *AtCBF3*-R | TTTGAAATGTTCCGAGCCAA |
| *AtCOR6.6*-F | AGAGACCAACAAGAATGCC |
| *AtCOR6.6*-R | TGTCCTTCACGAAGTTAACAC |
| *AtCOR15A*-F | GAACAAGCCTAGTGTCATCG |
| *AtCOR15A*-R | ATCATCCTCTGCTGTCTTGT |
| *AtCOR47*-F | GAAACCTCAAGAGACAACGA |
| *AtCOR47*-R | AGAAGAGCTGTTGGATCGG |
| *GAPDH-*F | TTTTTCAGCCATGGGCAA |
| *GAPDH*-R | TCATCGTTTTTCCACTGTCC |

Supplementary Table 5. Evaluation of PUB-ICE protein interactions via combined score in the STRING database. The string_id were formatted as species taxon ID and protein accession number, corresponds to entries retrievable in the NCBI database.

| ICE Protein | string_id | PUBs | string_id | combined_score |
| --- | --- | --- | --- | --- |
| OsICE1 | 39947.Q8H7N8 | OsPUB21 | 39947.Q2QU07 | 0.319 |
| TpICE1 | 57577.A0A2K3NL57 | TpPUB24 | 57577.A0A2K3L3G5 | 0.23 |
| TpICE1 | 57577.A0A2K3NL57 | TpPUB23 | 57577.A0A2K3LUR3 | 0.329 |
| TpICE1 | 57577.A0A2K3MXY9 | TpPUB24 | 57577.A0A2K3L3G5 | 0.223 |
| TpICE1 | 57577.A0A2K3MMZ2 | TpPUB24 | 57577.A0A2K3L3G5 | 0.215 |
| TpICE1 | 57577.A0A2K3N182 | TpPUB23 | 57577.A0A2K3LUR3 | 0.27 |
| GmICE1 | 3847.B8XJY5 | GmPUB23 | 3847.I1JGH3 | 0.219 |
| AtICE1 | 3702.Q9LSE2 | AtPUB26 | 3702.Q84TG3 | 0.208 |
| AtICE1 | 3702.Q9LSE2 | AtPUB26 | 3702.Q9FXA4 | 0.2 |
| AtICE1 | 3702.Q9LSE2 | AtPUB26 | 3702.Q9SVC6 | 0.158 |
| AtICE1 | 3702.Q9LSE2 | AtPUB26 | 3702.Q9LT79 | 0.371 |
| NaICE1 | 49451.A0A1J6J111 | NaPUB24 | 49451.A0A1J6JRB8 | 0.202 |
| CaICE1 | 4072.A0A2G3A5A8 | CaPUB1 | 4072.A0A2G2ZDB4 | 0.266 |
| CaICE1 | 4072.A0A2G3A2W1 | CaPUB1 | 4072.A0A2G2ZDB4 | 0.2 |
| SiICE1 | 4555.K3ZI10 | SiPUB21 | 4555.A0A368Q4C4 | 0.222 |
| SiICE1 | 4555.K3ZI10 | SiPUB21 | 4555.K3YPX2 | 0.253 |
| SiICE1 | 4555.K3ZI10 | SiPUB21 | 4555.K3Z3X4 | 0.207 |
| SiICE1 | 4555.K3ZI10 | SiPUB21 | 4555.K4A0Z2 | 0.221 |

**Supplementary Table 6. Relative expression levels of *MsPUB210* and *MsICE1* in multiple alfalfa cultivars.** The expression profiles of *MsPUB210* and *MsICE1* in 14 tested samples.

| Gene | Sample | Expression |
| --- | --- | --- |
| *MsPUB210* | Sample1 | 2.681172 |
| *MsICE1* | Sample1 | 0.76076 |
| *MsPUB210* | Sample2 | 3.911798 |
| *MsICE1* | Sample2 | 0.418042 |
| *MsPUB210* | Sample3 | 3.017045 |
| *MsICE1* | Sample3 | 0.406883 |
| *MsPUB210* | Sample4 | 4.315399 |
| *MsICE1* | Sample4 | 0.463813 |
| *MsPUB210* | Sample5 | 3.954 |
| *MsICE1* | Sample5 | 2.262364 |
| *MsPUB210* | Sample6 | 3.80969 |
| *MsICE1* | Sample6 | 0.642427 |
| *MsPUB210* | Sample7 | 6.608634 |
| *MsICE1* | Sample7 | 0.546464 |
| *MsPUB210* | Sample8 | 3.862747 |
| *MsICE1* | Sample8 | 1.238003 |
| *MsPUB210* | Sample9 | 4.920215 |
| *MsICE1* | Sample9 | 0.577454 |
| *MsPUB210* | Sample10 | 15.44 |
| *MsICE1* | Sample10 | 4.08 |
| *MsPUB210* | Sample11 | 20.56 |
| *MsICE1* | Sample11 | 5.16 |
| *MsPUB210* | Sample12 | 15.94 |
| *MsICE1* | Sample12 | 3.55 |
| *MsPUB210* | Sample13 | 15.91 |
| *MsICE1* | Sample13 | 4.67 |
| *MsPUB210* | Sample14 | 17.51 |
| *MsICE1* | Sample14 | 4.84 |


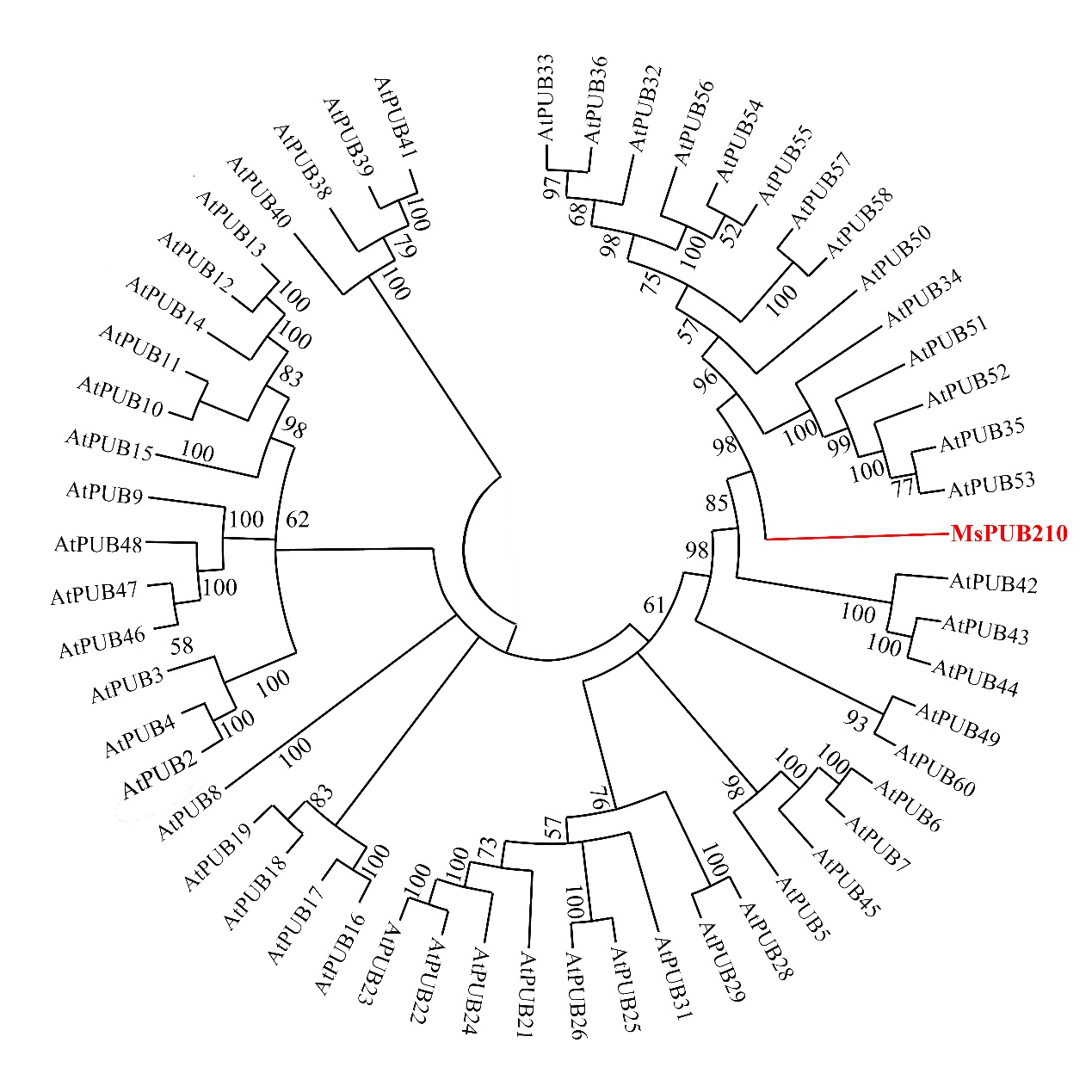


Supplementary Figure 2. Phylogenetic relationship of MsPUB210 and AtPUBs. Sequence alignment of 55 AtPUB proteins using 1000 bootstrap interactions, with the neighbor-joining (NJ) method to construct the tree. The MsPUB210 was highlighted with red, and the label of bootstraps were above the branch.


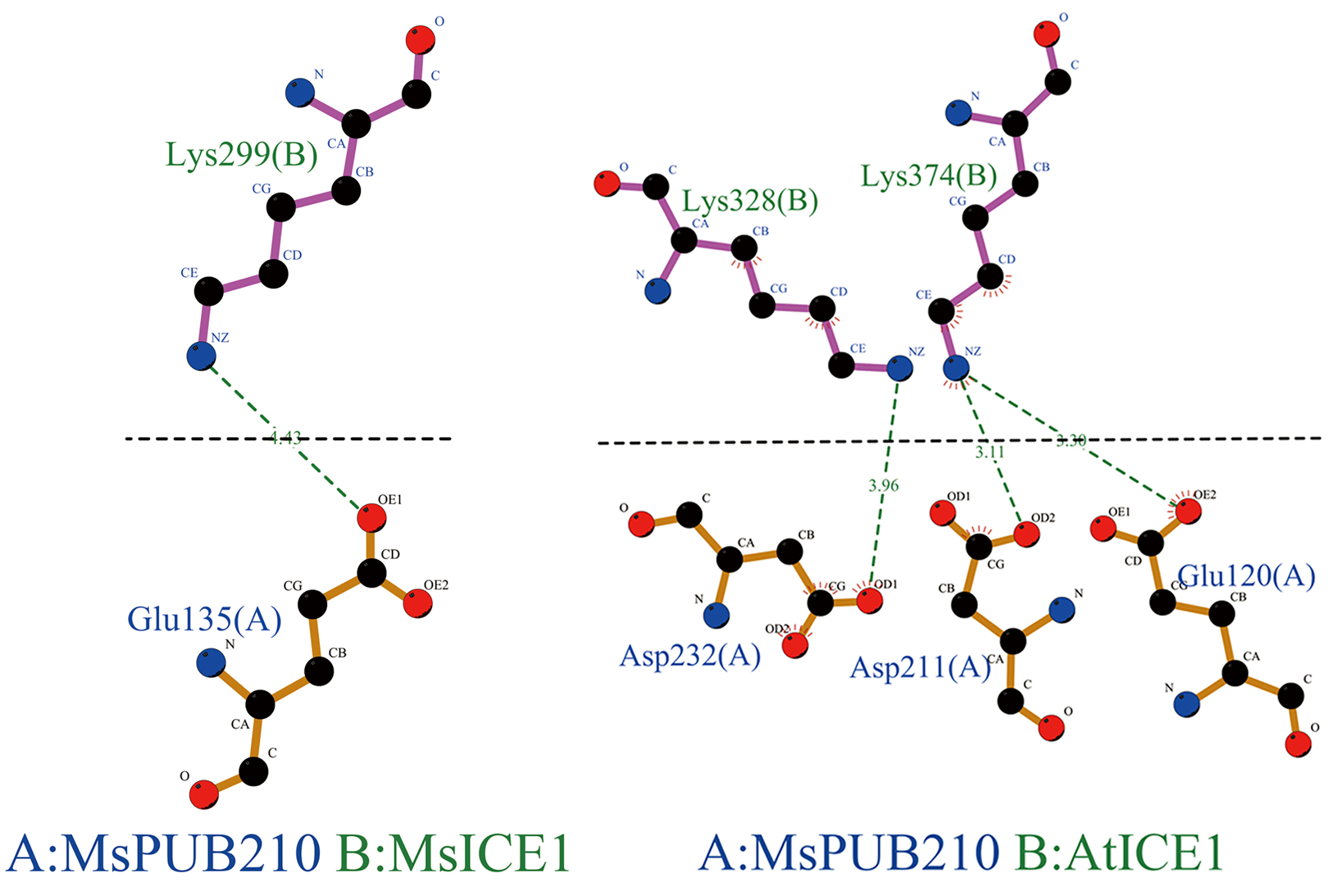


Supplementary Figure 3. 2D interaction diagrams of MsPUB210 with MsICE1 and AtICE1. Chain A (MsPUB210 protein) residues: blue, Chain B (MtICE1/AtICE1 protein) residues: green. Green dashed lines indicate hydrogen bonds or salt bridges. For MsPUB210–MsICE1 interaction, Lys299 (A, blue) interacts with Glu135 (B, green); for MsPUB210-AtICE1, Lys328/374 (A, blue) bind Asp232/Asp211/Glu120 (B, green).


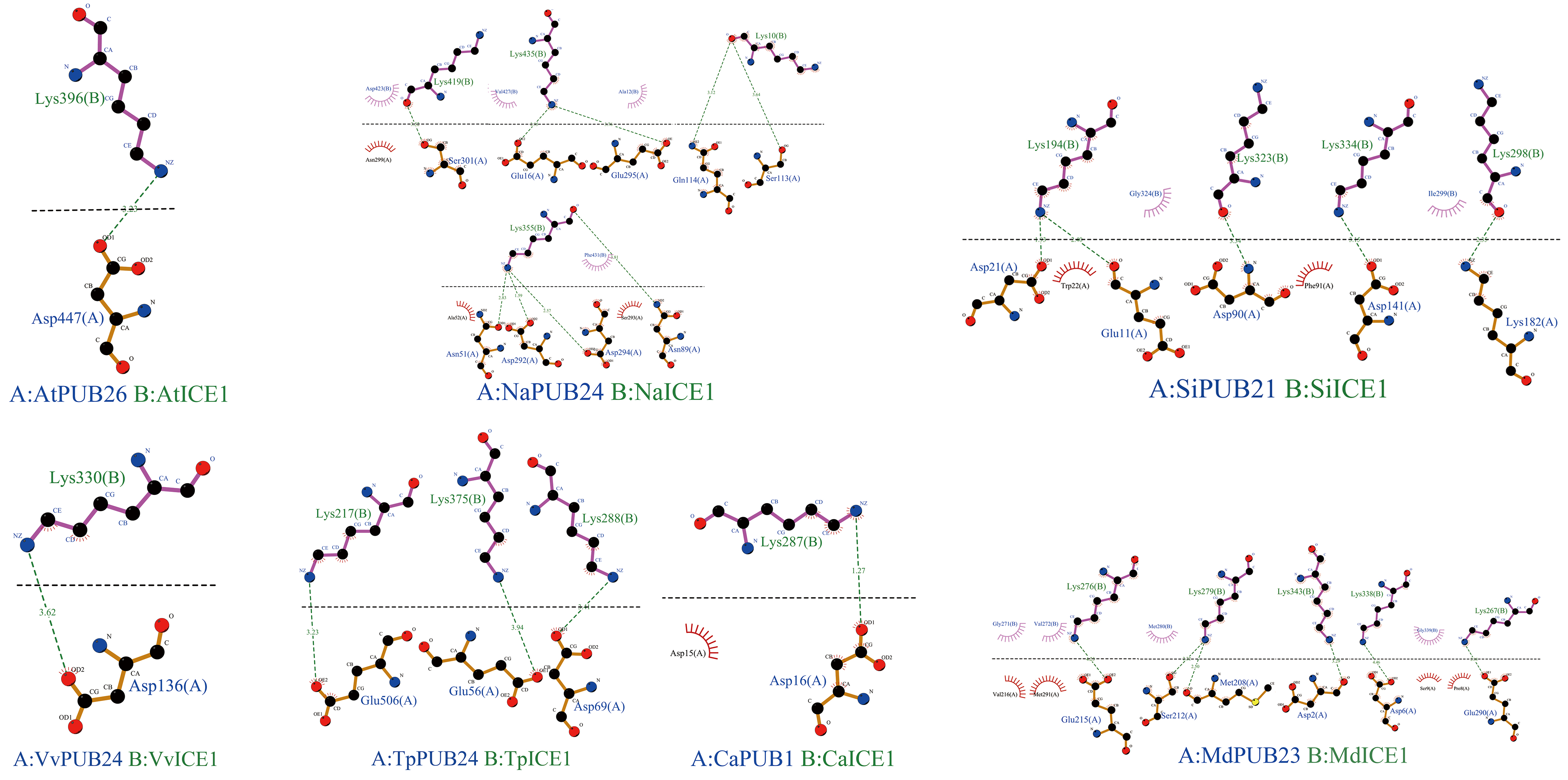


Supplementary Figure 4. 2D interaction diagrams of PUB–ICE pairs across multiple species. Chain A (PUB proteins) residues: blue, Chain B (ICE1 proteins) residues: green, with green dashed lines representing hydrogen bonds/salt bridges. In cross-species pairs (AtPUB26–AtICE1, NaPUB24–NaICE1, SiPUB21–SiICE1, VvPUB24–VvICE1, TpPUB24–TpICE1, CaPUB1–CaICE1, MdPUB23–MdICE1), green-labeled Lys residues in PUBs (Chain A) act as key interface residues, forming electrostatic interactions with blue-labeled Asp/Glu residues in ICEs (Chain B).


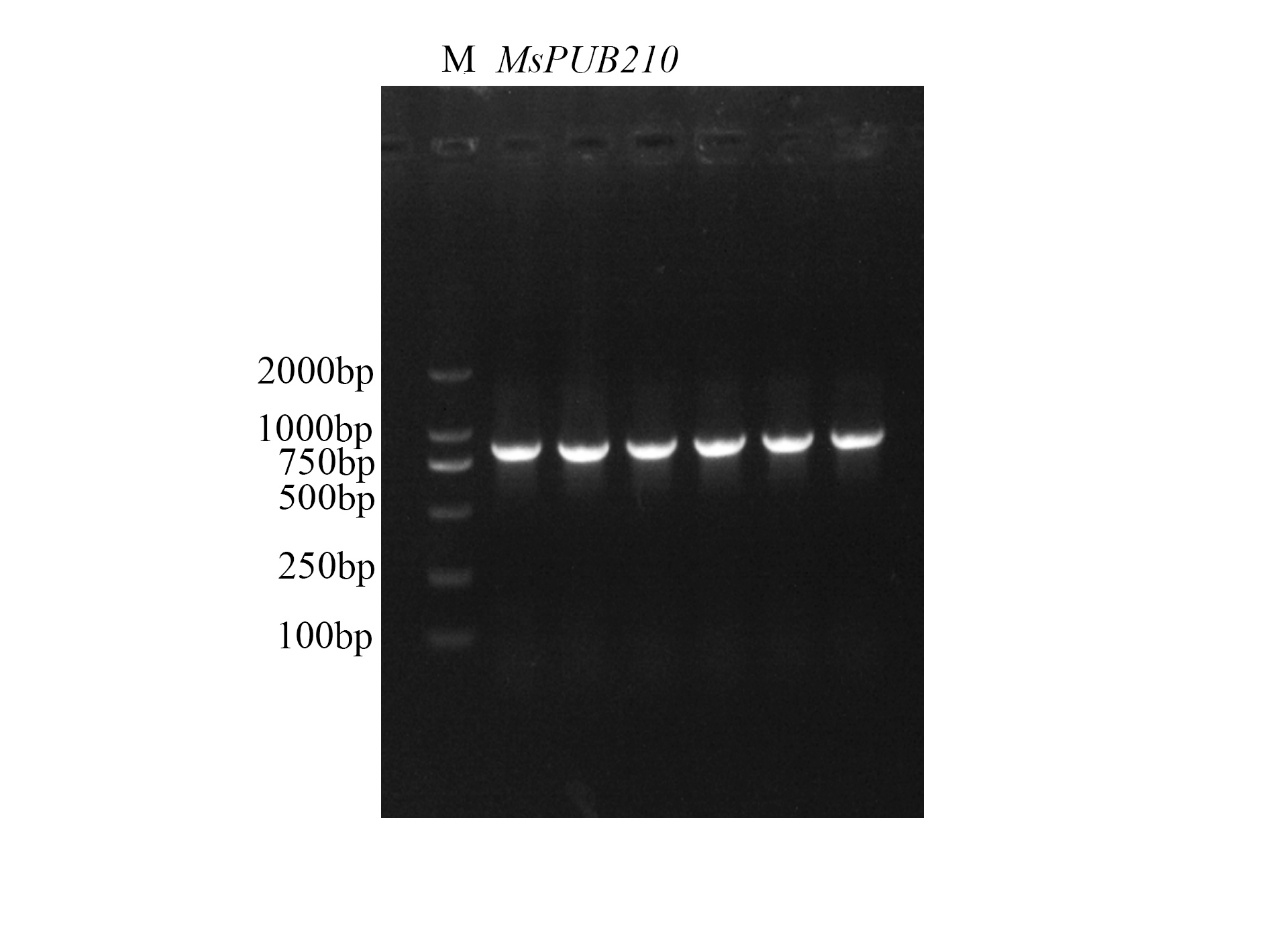


Supplementary Figure 5.PCR validation of *MsPUB210* amplification from *Medicago Sativa* cDNA. The full-length sequence of *MsPUB210* as template, amplified with gene-specific primers (*MsPUB210*-F and *MsPUB210*-R). Using 1.0% agarose gel electrophoresis to separate the product bands, an expected band size near 750 bp of marker was observed, ensuring successful amplification of the target gene fragment.
